# Supplementary material for: Modeling nonlinear and interaction effects of spatiotemporal and nongenetic factors improves prediction for complex traits
Source: Nat Commun. 2026 Aug 29;17:10333. doi: 10.1038/s41467-026-76154-7 (PMC13623873; doi:10.1038/s41467-026-76154-7)
Supplement: Supplementary file 1 [file 41467_2026_76154_MOESM14_ESM.pdf]

nature portfolio

Corresponding author(s): Melissa Gymrek

Last updated by author(s): May 16, 2026

Reporting Summary

Nature Portfolio wishes to improve the reproducibility of the work that we publish. This form provides structure for consistency and transparency in reporting. For further information on Nature Portfolio policies, see our Editorial Policies and the Editorial Policy Checklist.

Statistics

For all statistical analyses, confirm that the following items are present in the figure legend, table legend, main text, or Methods section.

n/a	Confirmed	
☐	☒	The exact sample size (*n*) for each experimental group/condition, given as a discrete number and unit of measurement
☐	☒	A statement on whether measurements were taken from distinct samples or whether the same sample was measured repeatedly
☐	☒	The statistical test(s) used AND whether they are one- or two-sided<br>*Only common tests should be described solely by name; describe more complex techniques in the Methods section.*
☐	☒	A description of all covariates tested
☐	☒	A description of any assumptions or corrections, such as tests of normality and adjustment for multiple comparisons
☐	☒	A full description of the statistical parameters including central tendency (e.g. means) or other basic estimates (e.g. regression coefficient) AND variation (e.g. standard deviation) or associated estimates of uncertainty (e.g. confidence intervals)
☐	☒	For null hypothesis testing, the test statistic (e.g. *F*, *t*, *r*) with confidence intervals, effect sizes, degrees of freedom and *P* value noted<br>*Give P values as exact values whenever suitable.*
☒	☐	For Bayesian analysis, information on the choice of priors and Markov chain Monte Carlo settings
☒	☐	For hierarchical and complex designs, identification of the appropriate level for tests and full reporting of outcomes
☐	☒	Estimates of effect sizes (e.g. Cohen's *d*, Pearson's *r*), indicating how they were calculated

Our web collection on statistics for biologists contains articles on many of the points above.

Software and code

Policy information about availability of computer code

Data collection	No software was used for data collection.
Data analysis	DeeperNull (developed here): https://github.com/gymrek-lab/DeeperNull v1.0.0<br>plink2 v2.00a2.3LM & v2.00a6LM<br>PRS-CS v1.1.0<br>snpnet BASIL (no version)<br>Python 3 with the following libraries:<br>matplotlib 3.10.8<br>numpy 2.2.6<br>pandas 2.3.3<br>pytorch-lightning 2.6.1<br>scikit-learn 1.7.2<br>scikit-posthocs 0.11.2<br>scipy 1.15.2<br>seaborn 0.13.2<br>shapiq 1.4.1<br>pytorch 2.10.0<br>torchmetrics 1.8.2<br>xgboost 3.2.0

For manuscripts utilizing custom algorithms or software that are central to the research but not yet described in published literature, software must be made available to editors and reviewers. We strongly encourage code deposition in a community repository (e.g. GitHub). See the Nature Portfolio guidelines for submitting code & software for further information.

Data

Policy information about availability of data

All manuscripts must include a data availability statement. This statement should provide the following information, where applicable:

- Accession codes, unique identifiers, or web links for publicly available datasets
- A description of any restrictions on data availability
- For clinical datasets or third party data, please ensure that the statement adheres to our policy

We used genetic, covariate, and phenotype datasets provided by UK Biobank under application number 46122 (see https://www.ukbiobank.ac.uk/use-our-data/apply-for-access).

Research involving human participants, their data, or biological material

Policy information about studies with human participants or human data. See also policy information about sex, gender (identity/presentation), and sexual orientation and race, ethnicity and racism.

Reporting on sex and gender	Sex was considered in the study design. Sex was always included as an input to the null and PGS models, as it is one of the most impactful covariates. We identified cases where sex interacted with other covariates in ways that substantially impacted phenotypes through null model interpretation methods. Findings apply to both sexes. Sex was determined by an agreement between genetic sex and self reported sex or sex in NHS records if no self-report was provided. Participants with a discordance were removed. Participants with sex chromosome aneuploidy were also removed. Source data cannot be shared, as it is from the UK Biobank. Gender was not used.
Reporting on race, ethnicity, or other socially relevant groupings	Participants in our paper were assigned to one of 5 ethnic groups. Membership in the White British group was assigned using the UK Biobank (UKB) field 22006. This field identified participants who self-identified as White British and have similar genetic ancestry based on principal components analysis. Membership in the other four groups was determined using self identified ethnicity (field 21000). The Black or Black British group contains participants who selected "Black or Black British" as their ethnic group, Asian or Asian British contains participants who selected "Asian or Asian British" or "Chinese" as their ethnic group, White and Black contains participants who selected "Mixed" for their ethnic group and then "White and Black Caribbean" or "White and Black African" for their ethnic background, and White and Asian contains participants who selected "Mixed" for their ethnic group and then "White and Asian" for their background.<br><br>Membership in an ethnic group was never used in modeling. As is common practice for this task and dataset, the null and PGS models were trained on members of the White British group. We did further analysis of the null models' ability to generalize to the non-White British participants.
Population characteristics	The population includes individuals aged 40-69 at the time of recruitment. The overall UK Biobank population is 54% female and 86% of participants lived in urban areas. We only included participants who were born in and currently live in England, Scotland, and Wales, as these are the participants with both birth and home location data available. Participants of White ethnicity make up 94.6% of the overall UK Biobank population, which is close to the matching the UK demographics at the time of recruitment.
Recruitment	The UK Biobank performed recruitment. People with NHS patient records aged 40-69 living within a reasonable distance from assessment centers were recruited. From all eligible people, a subset were mailed an invitation letter, with selection stratified by factors like age and sex and over-sampling where required. There is a healthy volunteer bias, in that participants are slightly healthier and wealthier than average for the UK population at the time of recruitment.
Ethics oversight	Ethics approval for this study is covered by the 2021 North West Multi-centre Research Ethics Committee approval renewal (REC reference: 21/NW/0157), which covers UK Biobank researchers without need for further approval. Access to UK Biobank was provided through application number 46122. Access to restricted location fields was further approved by the Access Management Team and the Data Analyst Team.

Note that full information on the approval of the study protocol must also be provided in the manuscript.

Field-specific reporting

Please select the one below that is the best fit for your research. If you are not sure, read the appropriate sections before making your selection.

☒ Life sciences ☐ Behavioural & social sciences ☐ Ecological, evolutionary & environmental sciences

For a reference copy of the document with all sections, see nature.com/documents/nr-reporting-summary-flat.pdf

Life sciences study design

All studies must disclose on these points even when the disclosure is negative.

Sample size	No sample size calculation was performed. Sample size was determined by the number of participants with data available for a given phenotype that passed the quality control requirements and had the ethnic background being used for the given training or evaluation.

Data exclusions

Non-White British participants were excluded from model training and the main model evaluation. This is common practice with this dataset due to the low number of non-White British participants, which can lead to poor generalization to non-White British people. We instead independently evaluate how our null models generalize to member of four non-White British ethnic backgrounds.

Otherwise, participants were excluded if they did not pass the standard QC checks for these types of analyses (i.e., unrelated, not outliers for missingness or heterozygosity, no sex chromosome aneuploidy or sex discordance) or if they were missing covariate data. Birth and home location were used as covariates in this study and data for them was only available if they were in England, Wales, or Scotland. As a result, people born outside these locations were excluded. For genotypes, only common variants that passed standard quality control checks were included.

Replication

To evaluate the reproducibility of our findings we evaluated the different modeling approaches across a number of phenotypes covering different categories of traits. 16 continuous phenotypes were used for the null and PGS model evaluation. These included anthropometric measurements, markers of lung, kidney, and liver function, blood pressure and lipid levels, and other blood-assay based biomarkers. 3 additional case-control phenotypes (asthma, depression, and diabetes) were also used for PGS evaluation.

Following best practices in machine learning, we used separate train, validation, and test set cohorts.

Randomization

Participants were randomly assigned to the training, validation, or test groups.

Blinding

This is not an interventional study, splits are purely for model training and evaluation, so blinding was not applicable.

Reporting for specific materials, systems and methods

We require information from authors about some types of materials, experimental systems and methods used in many studies. Here, indicate whether each material, system or method listed is relevant to your study. If you are not sure if a list item applies to your research, read the appropriate section before selecting a response.

Materials & experimental systems

n/a	Involved in the study
☒	☐ Antibodies
☒	☐ Eukaryotic cell lines
☒	☐ Palaeontology and archaeology
☒	☐ Animals and other organisms
☒	☐ Clinical data
☒	☐ Dual use research of concern
☒	☐ Plants

Methods

n/a	Involved in the study
☒	☐ ChIP-seq
☒	☐ Flow cytometry
☒	☐ MRI-based neuroimaging

Plants

Seed stocks

n/a

Novel plant genotypes

n/a

Authentication

n/a
