# Supplementary material for: Modeling nonlinear and interaction effects of spatiotemporal and nongenetic factors improves prediction for complex traits
Source: Nat Commun. 2026 Aug 29;17:10333. doi: 10.1038/s41467-026-76154-7 (PMC13623873; doi:10.1038/s41467-026-76154-7)
Supplement: Supplementary file 2 [file 41467_2026_76154_MOESM15_ESM.pdf]

nature portfolio

Peer Review File

Modeling nonlinear and interaction effects of spatiotemporal and nongenetic factors improves prediction for complex traits

Corresponding Author: Professor Melissa Gymrek

This file contains all reviewer reports in order by version, followed by all author rebuttals in order by version.

Version 0:

Reviewer comments:

Reviewer #1

(Remarks to the Author)
Modeling nonlinear and interaction effects of spatiotemporal and other non-genetic factors improves phenotypic prediction for complex traits

In standard practice, genome-wide association studies (GWAS) only adjust for a small set of non-genetic covariates, and these effects are typically modeled as linear. DeepNull proposed learning a flexible null model to relate non-genetic covariates to the phenotype, then including this model's predictions (along with the original covariates) as an additional adjustment in GWAS and downstream analyses. The present paper builds on DeepNull by proposing to include spatial and temporal information, such as location of birth and time-of-day/year, in the null model. Using approximately 300K subjects from the UK Biobank and a representative selection of continuous and binary traits, the authors evaluate three different classes of null model (linear, neural-network, gradient-boosted tree) across different covariate sets, then select the best for building polygenic scores. They observe that gradient-boosted trees provide a performant default architecture, and that the addition of spatiotemporal information in the null model improves prediction performance. A SHAP analysis is conducted to identify interactions, such as that between age and sex for LDL cholesterol, that linear modeling would have missed. Overall, the manuscript is clearly written and advances a simple, modular strategy for improving GWAS/PGS workflows by better incorporating non-linear spatiotemporal information.

1. Were the study participants filtered to unrelated individuals? If so, it would be helpful to state this explicitly. If not, please elaborate on whether relatedness was considered when splitting the data into train, validation, and test sets.

2. The methods note that the maximum allowable VIF was increased to 10^{6} to accommodate high correlation between the null model prediction and its component covariates. Would the polygenic score analyses be materially different if the null model prediction was selected into the polygenic score first then any highly correlated covariates were pruned out, for example by requiring that the R^{2} with the null model is below some threshold?

3. Day-of-year and time-of-day are currently encoded as integers. This is an inefficient positional encoding for linear models. As an alternative, you could consider the following. Let d denote day-of-year, and $\theta_{d} = 2\pi d/365.25$, then including $\sin(\theta_{d})$ and $\cos(\theta_{d})$ as two continuous, cyclic basis functions. Time-of-day can be encoded analogously.
Page 13 notes that vitamin D levels exhibit a sinusoidal pattern across the day-of-year, supporting the potential utility of the cyclic basis functions.

4. For location coordinates, using a spatially local surface may provide a significantly better basis than regarding spatial coordinates as having global linear effects. There are many ways to approach this. An unsupervised variant would be to represent the north-south (N) and east-west (E) axes using independent B-splines with a fixed number of knots (e.g. 10 placed at the quantiles), then representing position by the tensor product of these splines. A supervised variant could use the response (Y) to fit a 2D GAM/thin-plate spline of Y ~ g(N, E), then supply the prediction from this spatial model as a single additional input to the null prediction model. This latter approach is essentially a 2-stage DeepNull style analysis. Note that although a sufficiently complex null model could learn the spatial basis, or the temporal basis discussed in the last point,

providing an efficient basis makes the learning task significantly easier.

5. In the captions of Figures 2-4 and in the text as appropriate, please clarify on which data fold(s) (i.e., training, validation, or test) the evaluations are being performed.

6. Perhaps one important takeaway from Figure 3 is that although using the most expansive covariate set does not always lead to the best performance it seldom (perhaps never?) significantly underperforms the best covariate set.

7. In the Discussion, it may be worthwhile to connect this approach to the growing literature on prediction-based inference (PBI), which seeks to leverage predictions from machine-learning models to improve power while maintaining statistical validity (e.g., https://www.jmlr.org/papers/volume24/23-0896/23-0896.pdf, https://pubmed.ncbi.nlm.nih.gov/38872030/). DeepNull and the present strategy are examples of PBI insofar as they learn a flexible model of the phenotype then include the resulting phenotypic prediction in downstream analyses. This strategy differs from other approaches in that the predicted phenotype is used as a variance-reducing adjustment term rather than as a surrogate outcome (e.g., SynSurr).

(Remarks on code availability)
* The code is clearly written and documented. Including a script to generate synthetic example data for development and testing is particularly helpful.

* There is a directory that relates to UKB RAP workflows. It would be valuable to expand the README with details on how the proposed analysis end-to-end on RAP.

Reviewer #2

(Remarks to the Author)
The authors convincingly demonstrate that nonlinear modeling of covariates-particularly spatiotemporal features-can substantially improve phenotype prediction and downstream genetic analyses. I found the study to be well executed and the results impressive. However, I have the following comments and suggestions that I believe will further strengthen the manuscript:
1. Use of imputed data
UK Biobank whole-genome sequencing (WGS) data have been available since late 2023. Could the authors clarify why imputed genotype data were used instead of WGS data? A justification for this choice would be helpful, particularly given the potential advantages of WGS for rare variant analysis.
2. Quality control thresholds
The use of an imputation INFO score threshold of 0.3 appears relatively lenient. Could the authors justify this choice? I would strongly encourage presenting model comparisons under more stringent QC criteria (e.g., INFO ≥ 0.6 and MAF ≥ 0.05). Additionally, I did not find any mention of Hardy–Weinberg equilibrium filtering or genetic relatedness checks. Please clarify whether these steps were applied, and if not, provide a rationale.
3. Validation via simulation
The empirical results are compelling. Did the authors validate their findings using simulation studies? If so, I recommend including these results in the Supplementary Materials to strengthen the robustness of the conclusions.
4. Isolating PRS contribution
In real-data analyses, baseline covariates are typically included, which makes it difficult to isolate the unique contribution of polygenic risk scores (PRS). Is there a way to revise the presentation (e.g., nested models or incremental R^2) to more clearly separate the contribution of PRS from that of other covariates?
5. Generalizability beyond UK Biobank
Could the authors comment on the potential generalizability of the proposed method beyond UK Biobank? In particular, how might the approach perform in non-European populations, cross-ancestry settings, or smaller and less densely sampled cohorts?
6. Computational efficiency
Nonlinear models, particularly neural network–based approaches, can be computationally intensive. A brief comparison of computational time and memory requirements across models would be valuable for readers considering practical implementation.
7. Reproducibility and code availability
Code availability is essential for reproducibility. In several instances, the authors state that default settings were used, but many of the tools involved have multiple hyperparameters and pruning/threshold options. I strongly recommend providing the exact commands and scripts used for model training in a public GitHub repository.
8. Model selection strategy
In line 134, the authors mention fitting 2,304 models. Have the authors considered using a machine-learning–based strategy (e.g., automated hyperparameter optimization or model selection algorithms) to identify the optimal predictive model more efficiently?
9. Statistical comparison of prediction accuracy
In Figure 3, prediction accuracies (R^2) are compared across models. The authors could formally test differences between R^2 values using the r2redux R package (https://doi.org/10.1016/j.ajhg.2023.01.004), which would strengthen the statistical interpretation of these comparisons.
10. Comparison of classification performance
The authors analyzed three case–control traits and reported auROC values. Statistical comparison of auROC between models could be performed using the R2ROC package (https://github.com/mommy003/R2ROC) or DeLong's test.

Additionally, I recommend consistently using the term "AUC" instead of "auROC" throughout the manuscript.

(Remarks on code availability)

Reviewer #3

(Remarks to the Author)
This paper evaluates an approach for modeling non-linear covariate interactions in genetic studies with a particular focus on spatiotemporal covariates. It builds from the null model framework, introduced by DeepNull, where an auxiliary (null) model is trained on a set of covariates --- typically age, sex, and/or other standard covariates --- to predict a phenotype. The resulting predictions are then included as a new covariate for downstream analyses like running GWAS or computing a polygenic score (PGS). Prior work has demonstrated that the null model approach improves power to detect genetic associations and improves phenotype prediction from PGS.

This paper extends the null model approach through three main contributions. First, the authors demonstrate that XGBoost has better predictive performance on average than neural networks, like DeepNull, or standard linear models. Second, the authors show that use of spatiotemporal covariates like location and time improves phenotype predictions and PGS predictions over age and sex alone. Third, using XGBoost the authors show how non-linear null models can be used to find and interpret covariate interactions. Overall, I find the paper to be an interesting extension of the DeepNull approach. The experiments are well designed and demonstrate the key benefits of the author's approach.

I have the following comments:

[1] While the authors demonstrate that XGBoost has the best performance across phenotype-covariate pairs (Fig 2a), XGBoost is not always the best performing model per phenotype. A neural network was the top model for 12 of the 16 phenotypes evaluated (5 for the small neural network, 4 for the larger neural network, and 3 for DeepNull; lines 159-164). For downstream steps, an analyst may want to use the model with the best performance for their specific phenotype, even if XGBoost is better on average. It would be helpful to know, for those examples, how much better the neural network was and if that difference is significant. The authors my also consider spelling out the additional benefits of XGBoost over neural networks (e.g. ability to identify covariate interactions?) when setting up their comparison in the beginning of the Results. Together, I think these changes would make the justification for using XGBoost for the remaining experiments a bit stronger.

[2] The authors provide several examples where including time and location covariates improves phenotype prediction for null models. I would be curious to see if this improvement leads to additional GWAS signals, particularly because the authors state that improved genetic association testing is one of the main goals of their approach. Including one or two examples where using spatiotemporal variables leads to additional GWAS loci would bolster the contributions of the paper.

Minor comments:
[3] Line 111-113, numbering the model variations would make it easier to parse this sentence. For example, "1) age and sex,, … 2) home location, 3) birth location, etc."
[4] I would suggest annotating which model is used per phenotype in Fig 2b like in Fig 2a.
[5] Line 167-168: "The covariate sets including … showed top overall performance" – By what metric?
[6] Line 224 refers to 11/16 phenotypes with improved performance when using nonlinear null models and spatiotemporal features. It is a bit difficult to tell which phenotypes in Figure 3 this sentence is referring to. For some phenotypes the difference is obvious, for others the confidence intervals are close. Is it possible to annotate these on the figure itself?

(Remarks on code availability)
I downloaded and reviewed the code available on GitHub. The code is organized and easy to understand. It was nice to see that all the analysis scripts for experiments run on the UKB RAP were available. I was able to run the code to fit an XGBoost model on the provided example data. I have a few small suggestions to improve the code:
* Please list dependencies in a requirements.txt file or something similar.
* It would be helpful to either move the documentation from deeper_null/fit_model.py to the README.md or have a note in the README.md that the documentation is in that file.

Version 1:

Reviewer comments:

Reviewer #1

(Remarks to the Author)
The authors have responded thoughtfully to all comments from the last round of review. I congratulate them on their work.

(Remarks on code availability)

Reviewer #2

(Remarks to the Author)
can be accepted

(Remarks on code availability)
can be accepted

Reviewer #3

(Remarks to the Author)
The authors have addressed my comments.

(Remarks on code availability)

Open Access This Peer Review File is licensed under a Creative Commons Attribution 4.0 International License, which permits use, sharing, adaptation, distribution and reproduction in any medium or format, as long as you give appropriate credit to the original author(s) and the source, provide a link to the Creative Commons license, and indicate if changes were made.
In cases where reviewers are anonymous, credit should be given to 'Anonymous Referee' and the source.
The images or other third party material in this Peer Review File are included in the article's Creative Commons license, unless indicated otherwise in a credit line to the material. If material is not included in the article's Creative Commons license and your intended use is not permitted by statutory regulation or exceeds the permitted use, you will need to obtain permission directly from the copyright holder.
To view a copy of this license, visit https://creativecommons.org/licenses/by/4.0/

Overview

We thank the reviewers for their time and effort in evaluating our manuscript. Their feedback has led to substantial improvements in both the clarity and strength of our results. We highlight here the major areas addressed in our revision.

1. Additional analyses to strengthen conclusions: We added several new analyses requested by reviewers, including pairwise significance testing for PGS model comparisons (**Supplementary Table 8**), a comparison of null model vs. PGS contributions to overall performance (**Supplementary Figure 4**), GWAS loci counts with and without the spatiotemporal null (**Supplementary Table 7**), training cost and runtime comparisons across model classes (**Supplementary Table 4**), and an evaluation of cyclic basis function representations for temporal covariates (**Supplementary Table 3**).

2. Generalizability to non-White British participants: In response to Reviewer 2's question about generalizability, we evaluated how our linear and XGBoost null models, which were trained exclusively on White British individuals, performed on participants of non-White British self-reported ancestry (Black/Black British, Asian/Asian British, White+Black, White+Asian). As expected, our models generally performed worse on other ancestry groups not used in training. The decrease in performance varied substantially by group, from a 77.2% median decrease in R^2 for Black or Black British participants to a median 4.8% improvement for the White and Asian group with XGBoost null models. Notably, the XGBoost advantage over linear models was maintained in all four groups. These results are discussed in the updated Results and Discussion sections and presented in the new **Supplementary Table 5** and **Supplementary Figure 2**.

3. Bug fix in neural network evaluation: While investigating Reviewer 3's comment about per-phenotype model performance, we discovered that the neural network models were only saving predictions for the first batch of participants to the output file. After correcting this, the XGBoost models are now even more clearly the best performers, achieving the highest R^2 for all 16 phenotypes individually in addition to having the best aggregate performance. We additionally show that PGS models using XGBoost nulls outperform those using the best neural network null for all four phenotypes where a neural network was best in terms of MAE.

Responses to additional comments: Detailed responses to each of the reviewers comments are below. Reviewer comments are in black while responses are in blue. Revisions in the main text are indicated using track changes in MS Word. Finally, we have addressed all editorial comments to comply with *Nature Communication* formatting guidelines.

Reviewer #1 Comments

Modeling nonlinear and interaction effects of spatiotemporal and other non-genetic factors improves phenotypic prediction for complex traits

In standard practice, genome-wide association studies (GWAS) only adjust for a small set of non-genetic covariates, and these effects are typically modeled as linear. DeepNull proposed learning a flexible null model to relate non-genetic covariates to the phenotype, then including this model's predictions (along with the original covariates) as an additional adjustment in GWAS and downstream analyses. The present paper builds on DeepNull by proposing to include spatial and temporal information, such as location of birth and time-of-day/year, in the null model. Using approximately 300K subjects from the UK Biobank and a representative selection of continuous and binary traits, the authors evaluate three different classes of null model (linear, neural-network, gradient-boosted tree) across different covariate sets, then select the best for building polygenic scores. They observe that gradient-boosted trees provide a performant default architecture, and that the addition of spatiotemporal information in the null model improves prediction performance. A SHAP analysis is conducted to identify interactions, such as that between age and sex for LDL cholesterol, that linear modeling would have missed. Overall, the manuscript is clearly written and advances a simple, modular strategy for improving GWAS/PGS workflows by better incorporating non-linear spatiotemporal information.

We thank the reviewer for the encouraging words and helpful comments on our manuscript.

1. Were the study participants filtered to unrelated individuals? If so, it would be helpful to state this explicitly. If not, please elaborate on whether relatedness was considered when splitting the data into train, validation, and test sets.

We filtered to unrelated individuals using UKB field 22020, which indicates which individuals were used to compute the genetic principal components available as UKB field 22009. This set only includes individuals unrelated to the 3rd degree. The beginning of the null model evaluation section and the participant data section of Methods have been updated to make this clear. (**p. 18**)

2. The methods note that the maximum allowable VIF was increased to 10^{6} to accommodate high correlation between the null model prediction and its component covariates. Would the polygenic score analyses be materially different if the null model prediction was selected into the polygenic score first then any highly correlated covariates were pruned out, for example by requiring that the R^{2} with the null model is below some threshold?

The rationale for including both the null model prediction and the potentially highly correlated underlying covariates is included in the Results subsection *Nonlinear null models of covariates improve PGS performance* **(p. 9**). This is based on the section of the DeepNull paper (McCaw et al. 2022) titled *DeepNull's covariates should remain in the association model*. This section

argues that the underlying covariates should still be included to adjust for any associations between the covariates and genotypes. The null model prediction itself is insufficient to adjust for this since it only models the association between the covariates and the phenotype. They support this argument with simulations that show greater bias in estimated genetic effects when the underlying covariates are not included in the association model alongside the null model's prediction.

We evaluated the effect of removing the age and sex covariates for glycated haemoglobin and creatinine, respectively. These are the phenotypes for which the null prediction had the highest magnitude correlation with these covariates (r = 0.96 and r = 0.98). The covariate with the next largest magnitude correlation is day of year for vitamin D (r = 0.56). Removing the age covariate for glycated haemoglobin resulted in a small (0.2%) but not statistically significant increase in R^2. Removing the sex covariate for creatinine resulted in a small but statistically significant decrease in R^2. This is detailed in the *Polygenic score models* subsection of Methods, as this is where high correlations between the null prediction and covariates are discussed (**p. 21**).

3. Day-of-year and time-of-day are currently encoded as integers. This is an inefficient positional encoding for linear models. As an alternative, you could consider the following. Let d denote day-of-year, and $\theta_{d} = 2\pi d/365.25$, then including $\sin(\theta_{d})$ and $\cos(\theta_{d})$ as two continuous, cyclic basis functions. Time-of-day can be encoded analogously.
Page 13 notes that vitamin D levels exhibit a sinusoidal pattern across the day-of-year, supporting the potential utility of the cyclic basis functions.

We agree that the cyclic basis function representations, and feature engineering in general, would be better for modeling these covariates for linear models and could benefit other model classes. The downside to these approaches is that they require a priori knowledge. One advantage of using nonlinear null models, as our paper promotes, is that they can instead learn the best representations of features for a given phenotype from the data itself.

To evaluate the potential benefits of using cyclic basis functions for this application, we trained all nine null model variations with basis function representation inputs for the time covariates for three phenotypes (vitamin D, triglycerides, and systolic blood pressure). Time of day, month of year, and day of year were all represented with two additional features in the manner suggested by the reviewer. In aggregate, the linear models had the largest improvement in performance with the addition of the basis function features (median 7.7%), the neural network models had less than half of that percent improvement (median 3.0%), and the XGBoost models performed slightly worse (median 0.1%). Even with these improvements, the linear and neural network models using cyclic basis functions performed worse than the XGBoost models without them. Of the three phenotypes, vitamin D was the one where the performance was on average the closest when using the cyclic basis functions to the XGBoost nulls without the basis functions (5.9% worse for neural network nulls, 11.3% worse for linear nulls). These results are detailed in the *Evaluating null model performance on continuous phenotypes* subsection (**p. 6**) and **Supplementary Table 3**, which is a new addition.

4. For location coordinates, using a spatially local surface may provide a significantly better basis than regarding spatial coordinates as having global linear effects. There are many ways to approach this. An unsupervised variant would be to represent the north-south (N) and east-west (E) axes using independent B-splines with a fixed number of knots (e.g. 10 placed at the quantiles), then representing position by the tensor product of these splines. A supervised variant could use the response (Y) to fit a 2D GAM/thin-plate spline of Y ~ g(N, E), then supply the prediction from this spatial model as a single additional input to the null prediction model. This latter approach is essentially a 2-stage DeepNull style analysis. Note that although a sufficiently complex null model could learn the spatial basis, or the temporal basis discussed in the last point, providing an efficient basis makes the learning task significantly easier.

As with comment 3, we agree in principle that feature engineering can be beneficial, especially for the linear models. We chose to only evaluate the effect of feature engineering for the time features, due to their cyclical nature lending itself to a clean representation, as you suggested. This evaluation did not find that the basis functions led to overall better performances. We do not believe that additionally evaluating this for the location features would have an effect on our core conclusion: modeling nonlinear effects and interactions of these features improves results at the null and downstream levels.

5. In the captions of Figures 2-4 and in the text as appropriate, please clarify on which data fold(s) (i.e., training, validation, or test) the evaluations are being performed.

Text describing which data split is being used for each component of the plot has been added to the captions of **Figs. 2-4**, as well as all Supplementary Figures. The splits used to evaluate null performance, evaluate PGS performance, and to compute interactions are mentioned in the body of the text. We added a mention of PGS evaluation being on the held out test set to Results in addition to Methods.

6. Perhaps one important takeaway from Figure 3 is that although using the most expansive covariate set does not always lead to the best performance it seldom (perhaps never?) significantly underperforms the best covariate set.

We agree this is an important point! We added the following two sentences to the *Nonlinear null models of covariates improve PGS performance* section to clarify this point (**p. 11**):

Using a null model with all additional spatiotemporal covariates never resulted in an R^2 or MAE lower than the 95% CI of the best performing variation when principal components were not included in the null. When PCs were included in the null model's input, the only case of either metric being lower than the best model's confidence interval was R^2 for C-reactive protein.

7. In the Discussion, it may be worthwhile to connect this approach to the growing literature on prediction-based inference (PBI), which seeks to leverage predictions from machine-learning models to improve power while maintaining statistical validity (e.g.,

https://www.jmlr.org/papers/volume24/23-0896/23-0896.pdf, https://pubmed.ncbi.nlm.nih.gov/38872030/). DeepNull and the present strategy are examples of PBI insofar as they learn a flexible model of the phenotype then include the resulting phenotypic prediction in downstream analyses. This strategy differs from other approaches in that the predicted phenotype is used as a variance-reducing adjustment term rather than as a surrogate outcome (e.g., SynSurr).

We thank the reviewer for drawing attention to the prediction-based inference (PBI) literature. While our approach and PBI methods are similar in that they both train a model to predict a phenotype, they differ in three fundamental ways that we believe justify treating them as distinct strategies. First, the role of the prediction differs. With null models, the prediction is used as an input covariate in downstream association testing or predictive models, where it reduces residual variation by better modeling covariate effects and their interactions. In PBI, the prediction typically serves as a surrogate label for samples where the true value (the phenotype in this setting) is unknown.

Second, the mechanism by which power increases differs. Null covariate models improve power by reducing residual phenotypic variance. PBI methods improve power by increasing the effective sample size, as they allow the use of otherwise unlabeled samples.

Third, the approaches differ in their relationship to statistical validity. Null covariate models preserve valid inference because the predicted phenotype is used only as a covariate to adjust for nongenetic effects. PBI methods have validity problems if predictions are used directly, as it is not valid to interpret the association between the predictions and some other variables (e.g., genotypes) as the same as the true association with the phenotype. As a result, most of the focus in PBI is on methods to maintain statistical validity. For null covariate models, the focus is more on the form and inputs of the model.

Given these distinctions, we feel a detailed discussion of PBI would risk conflating two fundamentally different strategies. We appreciate the reviewer highlighting this connection, and we hope the above clarification addresses the concern.

Remarks on code availability: The code is clearly written and documented. Including a script to generate synthetic example data for development and testing is particularly helpful.
There is a directory that relates to UKB RAP workflows. It would be valuable to expand the REA DME with details on how the proposed analysis end-to-end on RAP.

The README has been expanded to cover the functionality of the various UKB RAP workflows, outline the use of the training and attribution scripts, list dependencies and tested versions, and otherwise points to docstrings for more information.

Reviewer #2 Comments

The authors convincingly demonstrate that nonlinear modeling of covariates-particularly spatiotemporal features-can substantially improve phenotype prediction and downstream genetic analyses. I found the study to be well executed and the results impressive. However, I have the following comments and suggestions that I believe will further strengthen the manuscript:

We thank the reviewer for careful reading and helpful suggestions.

1. Use of imputed data
UK Biobank whole-genome sequencing (WGS) data have been available since late 2023. Could the authors clarify why imputed genotype data were used instead of WGS data? A justification for this choice would be helpful, particularly given the potential advantages of WGS for rare variant analysis.

We agree WGS would be beneficial for rare variant analysis. However, our analyses focus exclusively on common variants (MAF ≥1%, following Collister et al. 2022). The use of imputed genotype data for common variants is common practice in PGS development, in part because PGS methodology tolerates tagging variants if they maintain predictive value, rather than requiring identification of truly causal variants.

Our primary research question concerns the impact of nongenetic factors on downstream genetic analyses. We therefore do not expect that using WGS data would meaningfully alter our conclusions regarding the impact of nongenetic factors on PGS or null model performance. The substantial computational burden (estimated >15,000 GBP) further supports maintaining our current approach.

We have clarified the use of common imputed variants in the Results (**p. 9**) in addition to in Methods sections.

2. Quality control thresholds
The use of an imputation INFO score threshold of 0.3 appears relatively lenient. Could the authors justify this choice? I would strongly encourage presenting model comparisons under more stringent QC criteria (e.g., INFO ≥ 0.6 and MAF ≥ 0.05). Additionally, I did not find any mention of Hardy–Weinberg equilibrium filtering or genetic relatedness checks. Please clarify whether these steps were applied, and if not, provide a rationale.

Our genotype quality control pipeline primarily followed Collister et al. 2022 (referred to in our Methods section), a widely used guide for GWAS and PRS analyses in UK Biobank. We address each point in turn and have added clarification to the Methods section about the rationale for these choices.

INFO score threshold. We used a threshold of 0.3, consistent with the recommendation in Bycroft et al. (2018) cited by Collister et al. (2022). Bycroft et al. (2018) notes that an information score of α in a sample of M individuals is approximately equivalent to perfectly observed genotypes in a sample of αM individuals, and that a threshold of 0.3 or possibly lower should yield good power to detect association given UKB's large sample size. With our training set sizes ranging from 221,748 to 254,008, even at this threshold the effective sample size is at least 66,524 to 76,202. Since the focus of our paper is on the impact of nongenetic factors rather than on optimizing genotype quality control, we do not expect that a more stringent threshold would materially change our conclusions.

MAF threshold. We used a MAF threshold of 0.01, a standard threshold in the field for excluding rare variants from common variant analyses, which is also used in Collister et al. (2022) to define common variants.

Hardy-Weinberg equilibrium. HWE filtering was applied upstream by UK Biobank as part of their QC pipeline. Variants within each genotyping batch were tested for HWE among individuals of homogeneous European ancestry, and those violating the assumption were set to missing prior to imputation. Imputation was then performed using IMPUTE4, a reimplementation of IMPUTE2 (Bycroft et al. 2018; Howie et al. 2009). As Collister et al. (2022) note, HWE is an assumption of the IMPUTE2 imputation method, and it may therefore not be appropriate to test whether the resulting imputed variants conform to HWE. We therefore deemed additional post-imputation HWE filtering unnecessary. We now discuss this choice in the *Genotype data* section of Methods.

Relatedness. As described in our response to Reviewer 1's first comment, participants were filtered to unrelated individuals (up to the 3rd degree) using UKB field 22020. The beginning of the null model evaluation section and the participant data section of Methods have been updated to make this clear (**p. 18**).

3. Validation via simulation
The empirical results are compelling. Did the authors validate their findings using simulation studies? If so, I recommend including these results in the Supplementary Materials to strengthen the robustness of the conclusions.

The DeepNull paper (McCaw et al. 2022), which introduces the nonlinear null model, uses simulations to verify its core beneficial properties: association and predictive power increase when there are nonlinear covariate effects or interactions without losing power when there are only linear effects. We expand on this briefly now in the introduction on **p. 2**. The results of these simulations should be valid for all of our nonlinear null models, as they are the result of moving from a linear model to a nonlinear model which can capture the nonlinear and interaction effects. Our paper instead focuses on two additions: the impact of adding spatiotemporal covariates and our ability to identify important covariate interactions. It would be straightforward to create simulations where the additional covariates are important or where there are impactful interactions to detect. However, the question we were focused on was if these things would be

true and useful with the real data with the actual magnitudes of effects from these factors. We therefore focused on demonstrating these effects in real data.

4. Isolating PRS contribution
In real-data analyses, baseline covariates are typically included, which makes it difficult to isolate the unique contribution of polygenic risk scores (PRS). Is there a way to revise the presentation (e.g., nested models or incremental R^2) to more clearly separate the contribution of PRS from that of other covariates?

We thank the reviewer for raising this point. We have added a new **Supplementary Fig. 4** which shows the R^2 attributable to covariates alone (via the null model) versus the incremental additional contribution of the PGS, allowing readers to directly compare these components. It shows the relative performance of the baseline linear null and BASIL PGS models compared to the null and PGS versions with XGBoost nulls and spatiotemporal features for the continuous phenotypes. For the case-control phenotypes, there is a similar comparison without the linear null, as we did not evaluate these models elsewhere. In the *Nonlinear null models of covariates improve PGS performance* section, we now discuss the relative performance of null and PGS models for continuous and case-control phenotypes (**p. 11 & 12**).

5. Generalizability beyond UK Biobank
Could the authors comment on the potential generalizability of the proposed method beyond UK Biobank? In particular, how might the approach perform in non-European populations, cross-ancestry settings, or smaller and less densely sampled cohorts?

We agree that generalizability to non-European and less well sampled populations is an important consideration and thus decided to include an evaluation of null model performance on non-White British participants. We now look at how linear and XGBoost null models, which were trained in White British individuals, perform for participants that self-identified as Black or Black British, Asian or Asian British, White and Black, or White and Asian and discuss these results in the *Evaluating null model performance on continuous phenotypes* section (**p. 7**). Additionally, we've modified the Discussion section and added **Supplementary Table 5** and **Supplementary Fig. 2** to cover these results.

6. Computational efficiency
Nonlinear models, particularly neural network–based approaches, can be computationally intensive. A brief comparison of computational time and memory requirements across models would be valuable for readers considering practical implementation.

We agree that this would make for a more complete comparison and be useful in practice. We focused on the difference in costs for training the null models, as this is where the null model class selected will make a difference. **Supplementary Table 4** is new and contains the runtime, AWS instance type, and cost of training for all of the model variations with each covariate set for HDL cholesterol. It additionally includes the average costs across all covariate options for all model variations and model classes (i.e. linear, neural network, and XGBoost) for HDL-C. The

Evaluating null model performance on continuous phenotypes section now includes a paragraph discussing the relative runtimes and costs (**p. 6**).

7. Reproducibility and code availability
Code availability is essential for reproducibility. In several instances, the authors state that default settings were used, but many of the tools involved have multiple hyperparameters and pruning/threshold options. I strongly recommend providing the exact commands and scripts used for model training in a public GitHub repository.

Code and workflows used for model training and the other analyses have been available in the public GitHub repository, which was referenced in the *Code availability* section of the manuscript. This repository has been moved to the lab GitHub and is now available at https://github.com/gymrek-lab/DeeperNull. The README has been expanded, as described in the responses to reviewer 1's final comment and reviewer 3's last two comments. The *Code availability* section now describes what is in the repository.

8. Model selection strategy
In line 134, the authors mention fitting 2,304 models. Have the authors considered using a machine-learning–based strategy (e.g., automated hyperparameter optimization or model selection algorithms) to identify the optimal predictive model more efficiently?

Our approach is in essence a hyperparameter grid search structured to allow comparisons along the model class and covariate set dimensions, which were our primary axes of interest. This allowed us to evaluate the relative performances of these different options in a more complete manner. In practice, we found that including all covariates often resulted in the best PGS performance and practically never significantly underperformed the best covariate option. Therefore, in general it is not required to do such an exhaustive model search in practice. We highlight this in the text, as mentioned in our response to reviewer 1's comment 6.

We considered more extensive automated hyperparameter optimization for the specific hyperparameters of each model class (instead of evaluating three set variations) but decided against it for several reasons. First, the gains would likely be small, as the dominant factor in our results was the choice of model class and covariate set rather than fine-tuning within a class. Second, it would substantially increase computational costs, particularly for the neural network models, which already cost more than 20 times more to train than the XGBoost models (Supplementary Table 5). Third, the large differences in training time and search space across model classes would make it difficult to ensure a fair comparison, as a fixed optimization budget would allow far more thorough search over cheaper models like XGBoost than over neural networks. Finally, it could make the approach less user friendly for practitioners without a general ML background, who are a key audience for this work.

9. Statistical comparison of prediction accuracy
In Figure 3, prediction accuracies (R^2) are compared across models. The authors could formally test differences between R^2 values using the r2redux R package

(https://doi.org/10.1016/j.ajhg.2023.01.004), which would strengthen the statistical interpretation of these comparisons.

In addition to confidence intervals on R2 values, we now also include pairwise significance testing between the baseline PGS approach and all alternatives (new Supplementary Table 7). These results are discussed in the *Nonlinear null models of covariates improve PGS performance* section. We did not use the r2redux package, since several assumptions are violated by our PGS models (phenotypes are not adjusted for non-genetic nor environmental factors, genotypes are not standardized, and the PGS model is not purely sum of linear contributions of genotypes). Instead we used Fisher-Pitman tests (Bestgen 2022; Berry et al. 2002) for comparing all PGS variations against the standard approach, comparing PGS models using neural network or XGBoost based nulls, and evaluating if covariates highly correlated with the null prediction should be removed from the PGS input.

10. Comparison of classification performance
The authors analyzed three case–control traits and reported auROC values. Statistical comparison of auROC between models could be performed using the R2ROC package (https://github.com/mommy003/R2ROC) or DeLong's test. Additionally, I recommend consistently using the term "AUC" instead of "auROC" throughout the manuscript.

We have changed from using "auROC" to "AUC". As with comment 9, we now include p-values for a measure of significance but focus on the effect sizes and confidence intervals. We used Fisher-Pitman tests for the case-control phenotypes as well for consistency (and the ability to compare average precision as well as AUC).

Reviewer #3 Comments

This paper evaluates an approach for modeling non-linear covariate interactions in genetic studies with a particular focus on spatiotemporal covariates. It builds from the null model framework, introduced by DeepNull, where an auxiliary (null) model is trained on a set of covariates --- typically age, sex, and/or other standard covariates --- to predict a phenotype. The resulting predictions are then included as a new covariate for downstream analyses like running GWAS or computing a polygenic score (PGS). Prior work has demonstrated that the null model approach improves power to detect genetic associations and improves phenotype prediction from PGS.

This paper extends the null model approach through three main contributions. First, the authors demonstrate that XGBoost has better predictive performance on average than neural networks, like DeepNull, or standard linear models. Second, the authors show that use of spatiotemporal covariates like location and time improves phenotype predictions and PGS predictions over age and sex alone. Third, using XGBoost the authors show how non-linear null models can be used to find and interpret covariate interactions. Overall, I find the paper to be an interesting extension of the DeepNull approach. The experiments are well designed and demonstrate the key benefits of the author's approach.

We thank the reviewer for the helpful comments below which helped us to improve the manuscript.

I have the following comments:

[1] While the authors demonstrate that XGBoost has the best performance across phenotype-covariate pairs (Fig 2a), XGBoost is not always the best performing model per phenotype. A neural network was the top model for 12 of the 16 phenotypes evaluated (5 for the small neural network, 4 for the larger neural network, and 3 for DeepNull; lines 159-164). For downstream steps, an analyst may want to use the model with the best performance for their specific phenotype, even if XGBoost is better on average. It would be helpful to know, for those examples, how much better the neural network was and if that difference is significant. The authors my also consider spelling out the additional benefits of XGBoost over neural networks (e.g. ability to identify covariate interactions?) when setting up their comparison in the beginning of the Results. Together, I think these changes would make the justification for using XGBoost for the remaining experiments a bit stronger.

We thank the reviewer for this helpful comment. While looking into this, we discovered that our neural network models were only being evaluated on the first batch of participants, as they were only saving predictions for the first batch to the output file. The *Evaluating null model performance on continuous phenotypes* section has been updated to reflect this, and the XGBoost models are now more clearly the best performers both in aggregate and as the best per phenotype. The neural networks likely achieved as many top results in the original analysis because the smaller evaluation subset increased score variance. XGBoost models now have the best individual performance for all phenotypes in terms of R^2 as well as the best aggregate performance (**Fig. 2**). In terms of MAE, XGBoost models have the best aggregate performance and are best for 12/16 phenotypes (**Supplementary Fig. 1**). For the four phenotypes where a neural network was the best individual performer in terms of MAE, we now compare the performance of downstream PGS models with the best performing neural network or an XGBoost null with all covariates. We find the BASIL and PRS-CS PGS model with an XGBoost null always outperform those that use the neural network (**Supplementary Table 9**).

Additionally, we now look at the training costs and runtimes for the different classes of null models (**Supplementary Table 4; p. 6**). For HDL cholesterol, neural networks cost on average more than 23 times what it costs for an XGBoost model. We believe the greater difference in null performance, the full PGS evaluation, the much lower costs, and the desirable attribution method properties we discuss make the case for XGBoost models as the preferred null model class clear.

[2] The authors provide several examples where including time and location covariates improves phenotype prediction for null models. I would be curious to see if this improvement leads to additional GWAS signals, particularly because the authors state that improved genetic association testing is one of the main goals of their approach. Including one or two examples

where using spatiotemporal variables leads to additional GWAS loci would bolster the contributions of the paper.

We added **Supplementary Table 7**, which gives the total number of significant variants and loci with and without a spatiotemporal XGBoost null, as well as the number that are unique to each approach or common to both. We found an increased number of significant independent loci for 11/16 phenotypes when using the spatiotemporal XGBoost null. These results are discussed in *Nonlinear null models of covariates improve PGS performance* section in Results and the details have been added to the *Polygenic score models* section of Methods.

[3] Line 111-113, numbering the model variations would make it easier to parse this sentence. For example, "1) age and sex,, … 2) home location, 3) birth location, etc."

We agree. The change has been made.

[4] I would suggest annotating which model is used per phenotype in Fig 2b like in Fig 2a.

Fig. 2b (and the corresponding figure for MAE, **Supplementary Fig. 1b**) have been changed to now show the best performing model class for a phenotype-covariate set pair instead of using the marker type to distinguish between if PCs were included as input to the null model or not. We feel this is a more important result to focus on and include.

[5] Line 167-168: "The covariate sets including … showed top overall performance" – By what metric?

The sentence now clarifies that this is the case for both R^2 and MAE.

[6] Line 224 refers to 11/16 phenotypes with improved performance when using nonlinear null models and spatiotemporal features. It is a bit difficult to tell which phenotypes in Figure 3 this sentence is referring to. For some phenotypes the difference is obvious, for others the confidence intervals are close. Is it possible to annotate these on the figure itself?

In **Fig. 3** and **Supplementary Fig. 3** (the corresponding figure for PRS-CS) there are now asterisks over bars with a value outside the 95% confidence interval of the baseline age, sex, and no nonlinear null approach.

I downloaded and reviewed the code available on GitHub. The code is organized and easy to understand. It was nice to see that all the analysis scripts for experiments run on the UKB RAP were available. I was able to run the code to fit an XGBoost model on the provided example data. I have a few small suggestions to improve the code:
* Please list dependencies in a requirements.txt file or something similar.

The README now provides a list of dependencies for training and attribution and otherwise mentions using the Dockerfiles provided. A requirements.txt file was excluded to not break workflows that install the repository but have their requirements already installed in the docker container.

* It would be helpful to either move the documentation from deeper_null/fit_model.py to the README.md or have a note in the README.md that the documentation is in that file.

We agree the README was insufficient before. It now provides overviews, requirements, command line arguments, output files and the example usage for the training script and the script to get Shapley and interaction values. It also now contains descriptions of the UKB RAP workflows and otherwise points to docstrings for more information.
