# Supplementary material for: Modeling nonlinear and interaction effects of spatiotemporal and nongenetic factors improves prediction for complex traits
Source: Nat Commun. 2026 Aug 29;17:10333. doi: 10.1038/s41467-026-76154-7 (PMC13623873; doi:10.1038/s41467-026-76154-7)
Supplement: Supplementary file 3 [file 41467_2026_76154_MOESM1_ESM.pdf]

Supplementary Figures

Supplementary Figure 1

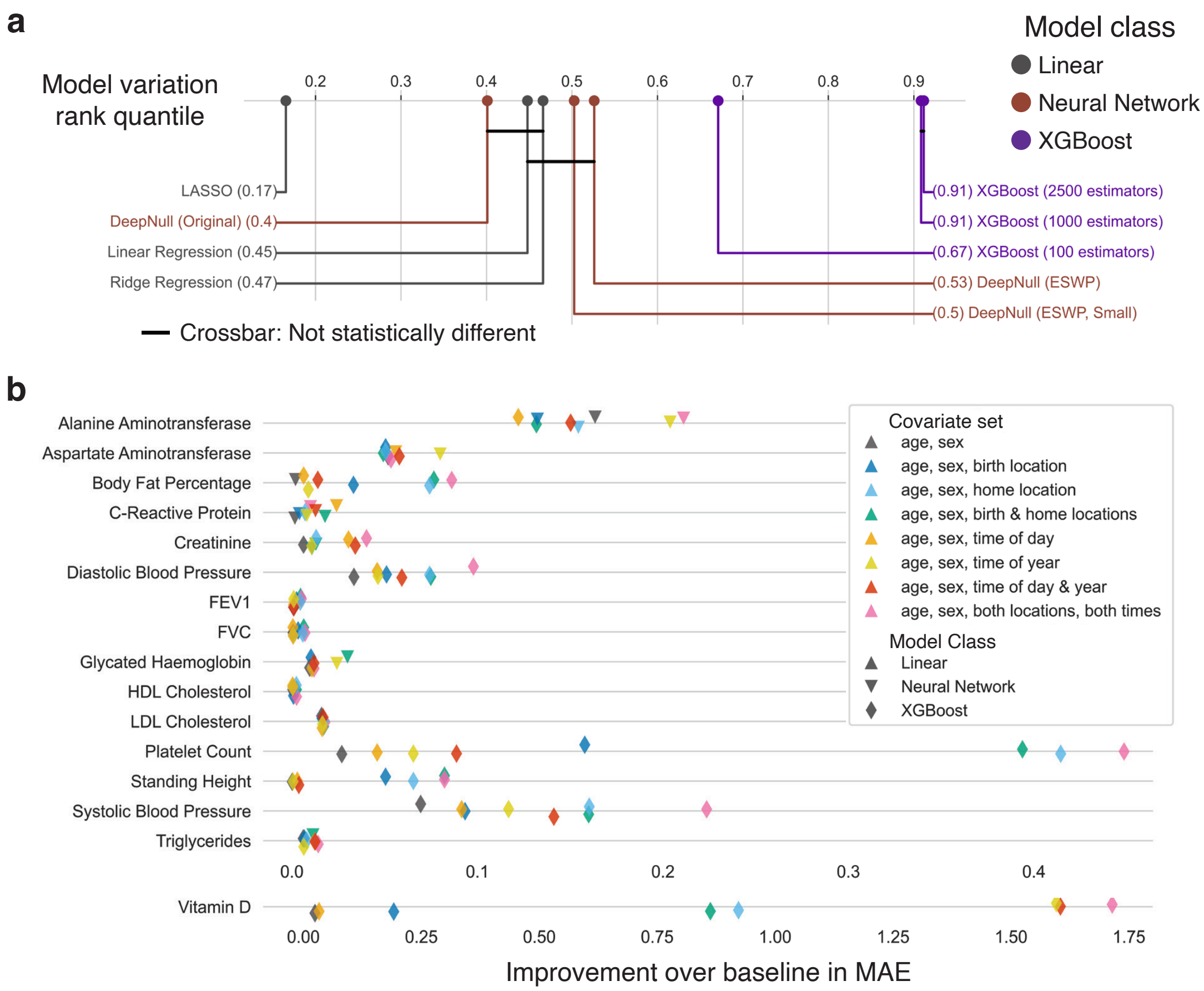

Null model evaluation results using mean absolute error (MAE).
a Model variation critical difference plot. The x-axis shows the quantile of a model variation's MAE rank across all pairs, where a higher quantile indicates better predictive power on average. The results in both panels of this figure result from five-fold cross validation using the training set. The horizontal crossbar connects model variates that were not statistically different with a Nemenyi post-hoc test α of 0.05 (following a significant Friedman test). Color indicates model class (gray=linear; red=neural network; purple=XGBoost). **b** Best null model improvement for each covariate set over the baseline approach. The x-axis shows the improvement in MAE of the best performing model variation for each covariate set we evaluated compared to a baseline linear model with age and sex alone. Marker shape indicates the class of the model variation. Color indicates the covariate set. The best performer for each covariate set with or without PCs included is shown. Scores underlying all components of this figure can be found in Supplementary Data 2 and the numbers of samples in the training, validation, and test sets for each trait are provided in Supplementary Data 1.

Supplementary Figure 2

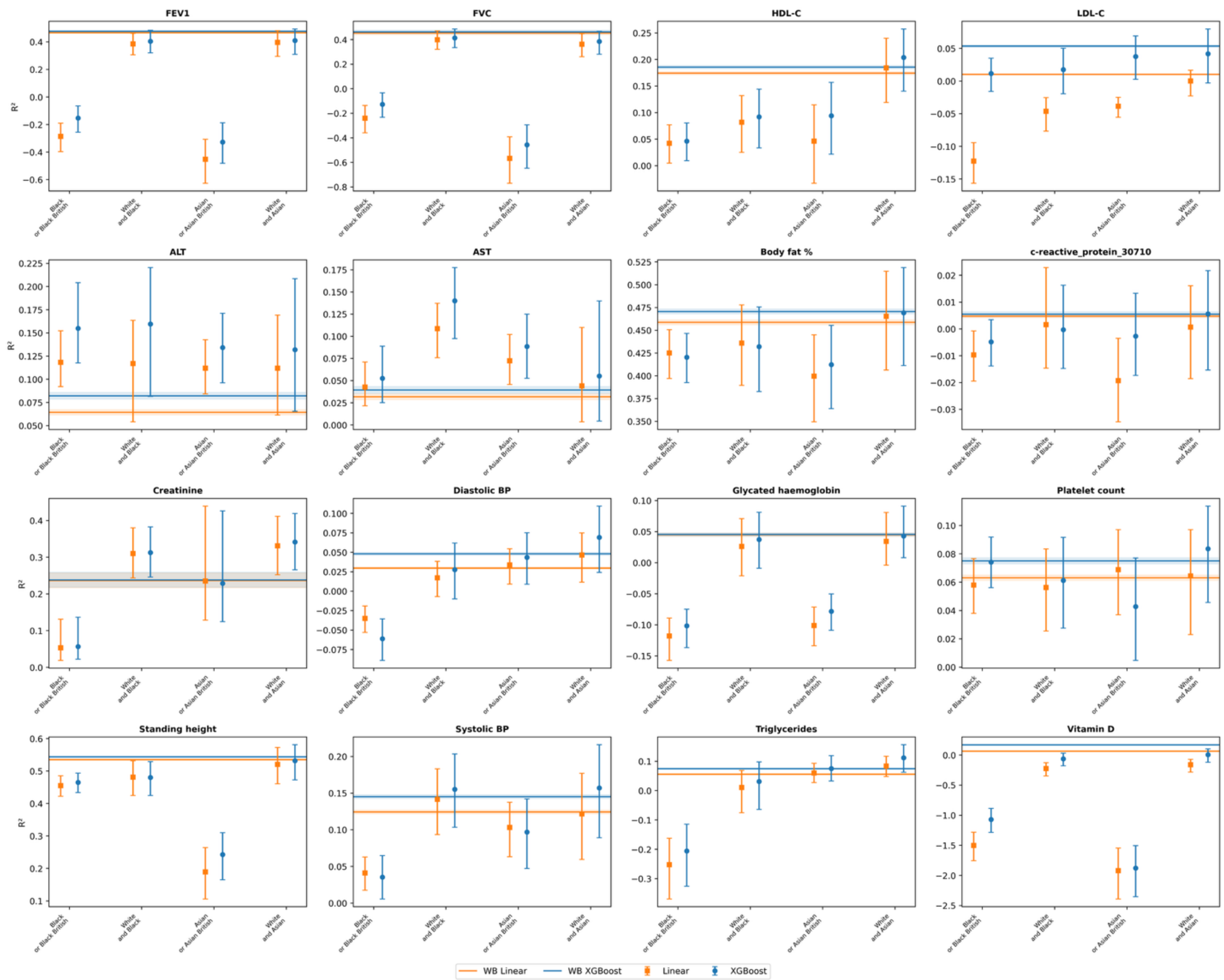

Null model evaluation for participants with self-reported non-White British ethnicity. Performance was evaluated for the aggregate best nonlinear null model variation (the largest XGBoost) and the best linear variation (ridge regression). Each subplot corresponds to a different phenotype. The y-axis represents the R^2 of the null models. Horizontal lines indicate performance on White British training set participants (*n* provided in Supplementary Data 1, 5-fold cross validation), provided as a baseline (source data values provided in Supplementary Data 2). The orange line is for the ridge regression model, the blue is for XGBoost, and their corresponding shaded regions represent a 95% confidence interval obtained with 10,000 bootstrapping iterations. The performances for samples with the non-White British self-reported ethnicities are shown with markers: orange squares for the ridge regression performance and blue circles for the XGBoost null. Their error bars show a 95% CI obtained in the same manner. The non-White British sample sizes (*n*), scores, and CIs are provided in Supplementary Data 5.

Supplementary Figure 3

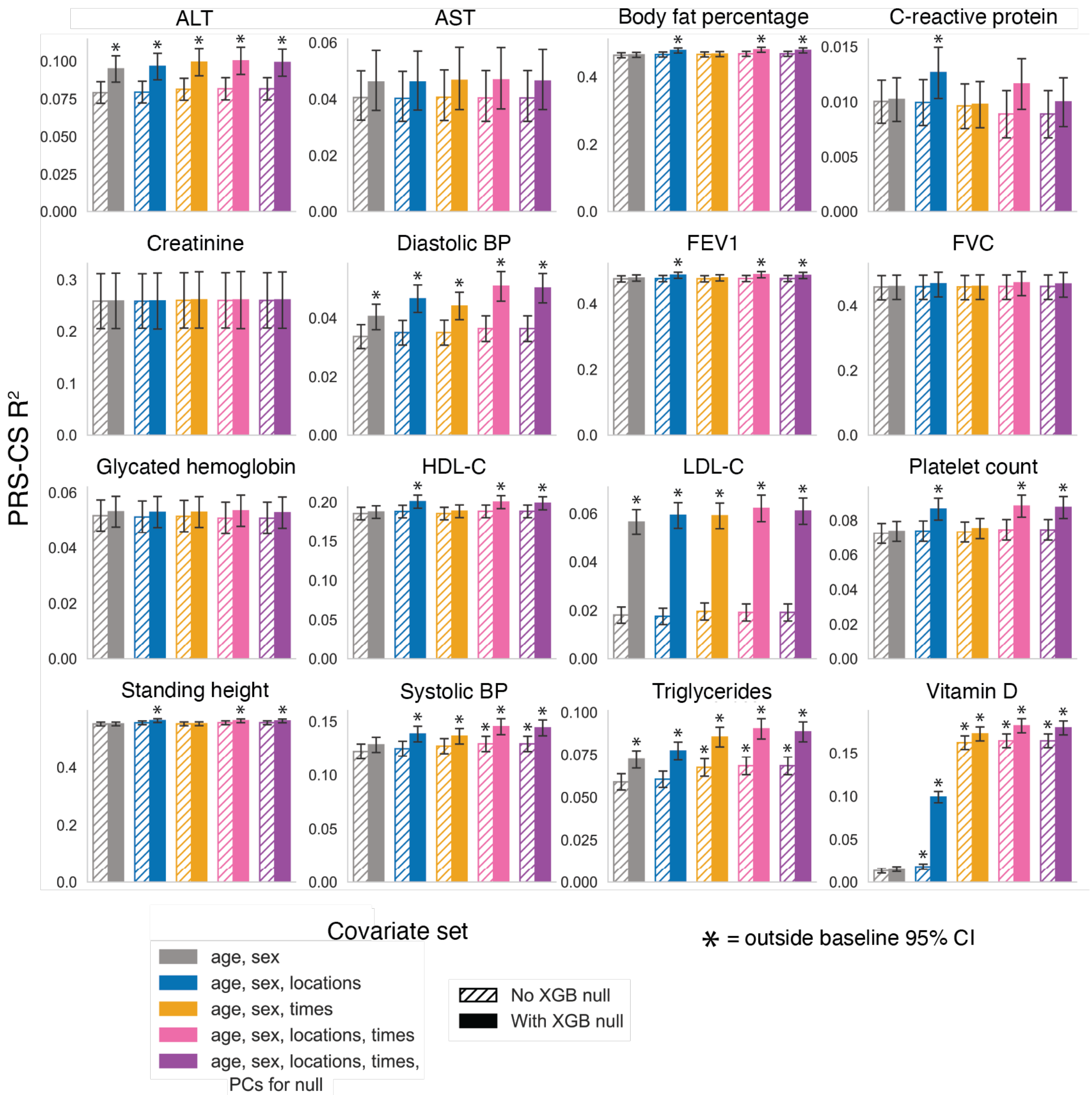

PRS-CS PGS model performance for continuous phenotypes. Performance for continuous phenotypes (source data in Supplementary Data 6). Bars show test set performance of PRS-CS PGS models, measured in R^2, for the 16 continuous phenotypes when the different covariate options were evaluated with and without a nonlinear null. Error bars show 95% bootstrapped confidence intervals (n = 100,000) around the test set R^2. For each covariate set, the hatched bar shows the R^2 when no nonlinear null was used and filled bars show performance when the nonlinear XGBoost null (XGB) was included. Asterisks above bars indicate that their value is greater than the 95% confidence interval (CI) of the baseline age, sex, and no null model approach. The pink and purple hashed bars are the same, as the only difference between the "age, sex, locations, times" and "age, sex, locations, times, PCs for null" covariate sets is whether the PCs are included as input to the null in addition to the other covariates. Train, validation, and test set sizes for all phenotypes are provided in Supplementary Data 1.

Supplementary Figure 4

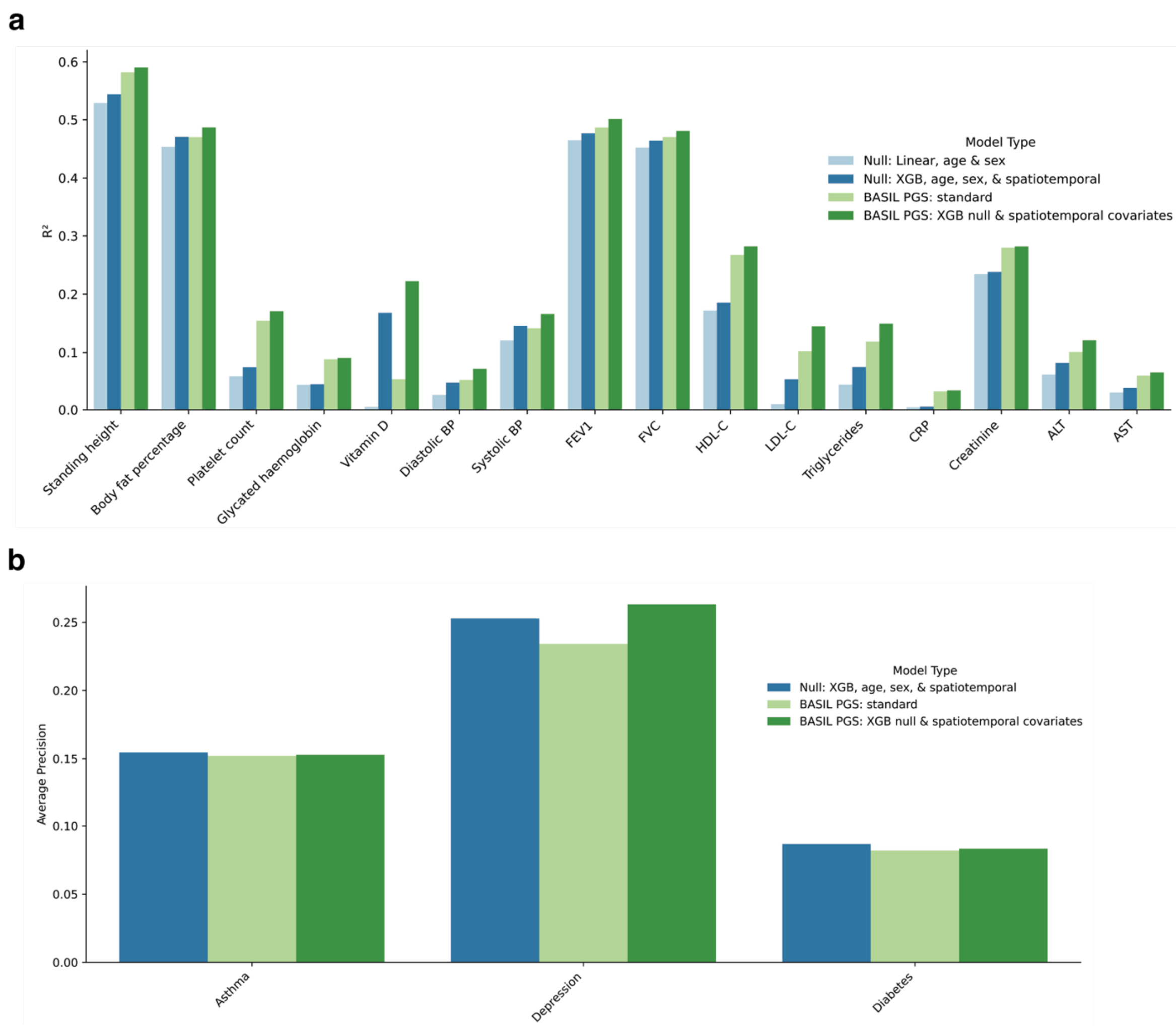

Comparison of null and PGS model performances. a Null model and BASIL PGS performance for continuous phenotypes. Blue bars represent the performance of the null models on the training set using 5-fold cross validation (source data provided in Supplementary Data 2) and green bars show BASIL PGS performance of the test set, both in terms of R^2 (source data for scores in Supplementary Data 6, sample sizes in Supplementary Data 1). The lighter blue bar is for a linear null using just age and sex as input, while the darker blue bar shows the performance of the largest XGBoost null using age, sex, and the additional spatiotemporal covariates. The lighter green bar is for the standard BASIL PGS model, in which just age, sex, and the genetic PCs are used as covariates with no null model. The darker green bar represents a BASIL PGS that adds the spatiotemporal covariates and the largest XGBoost null using age, sex, and the additional spatiotemporal covariates. **b** Null model and BASIL PGS performance for case-control phenotypes. The bars represent the performance of the various models in terms of average precision (source data provided in Supplementary Data 1, Supplementary Data 2, and Supplementary Data 6). The colors are as above, except there are no light blue bars, as we did not evaluate linear model performance for these phenotypes.

Supplementary Figure 5

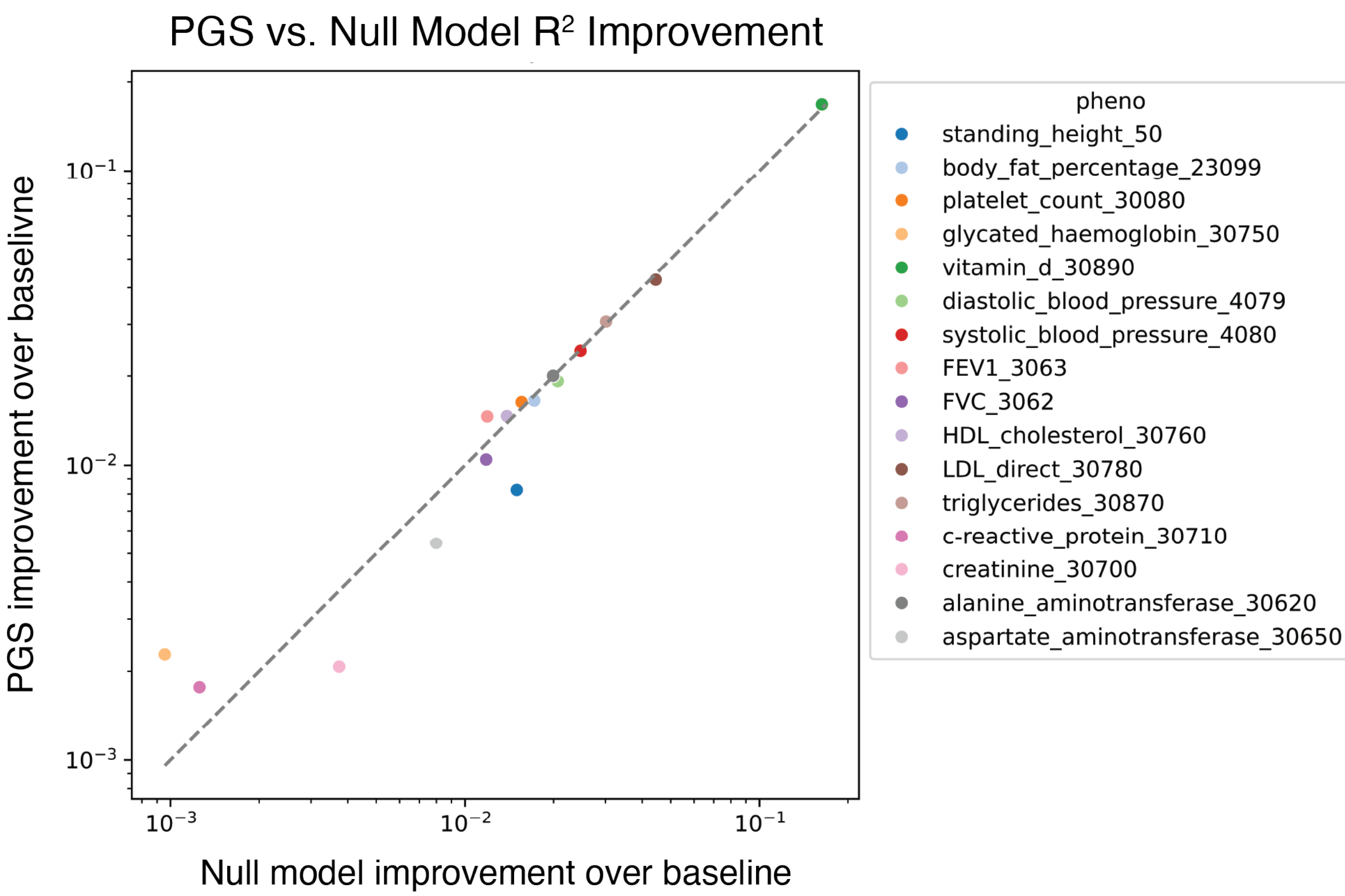

Correlation between null and PGS improvements over baseline with spatiotemporal covariates and nonlinear null models. The x-axis shows the improvement in R^2 of the largest XGBoost nonlinear null model using all the additional spatiotemporal covariates over the baseline approach (a linear regression null model using just age and sex) from five-fold cross validation with the training set (source data in Supplementary Data 1, Supplementary Data 2). The y-axis shows the test set improvement in test set R^2 of a BASIL PGS that uses the nonlinear null and additional spatiotemporal features over one that does not (source data in Supplementary Data 1, Supplementary Data 6). The Pearson correlation of these improvements is 0.9985 with a two-sided p-value of 5.4e-19.

Supplementary Figure 6

Supplementary Figure 6a

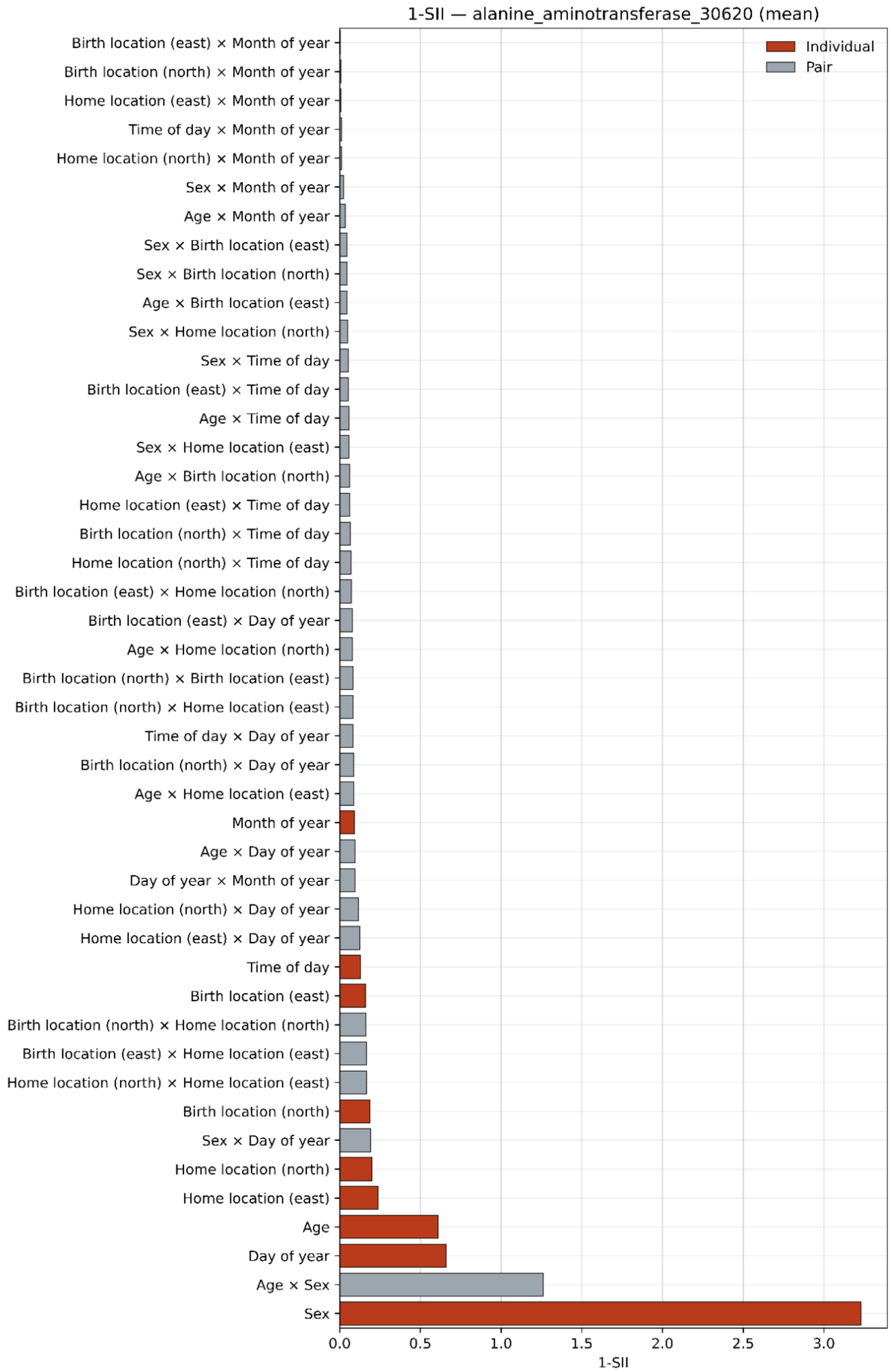

Supplementary Figure 6b

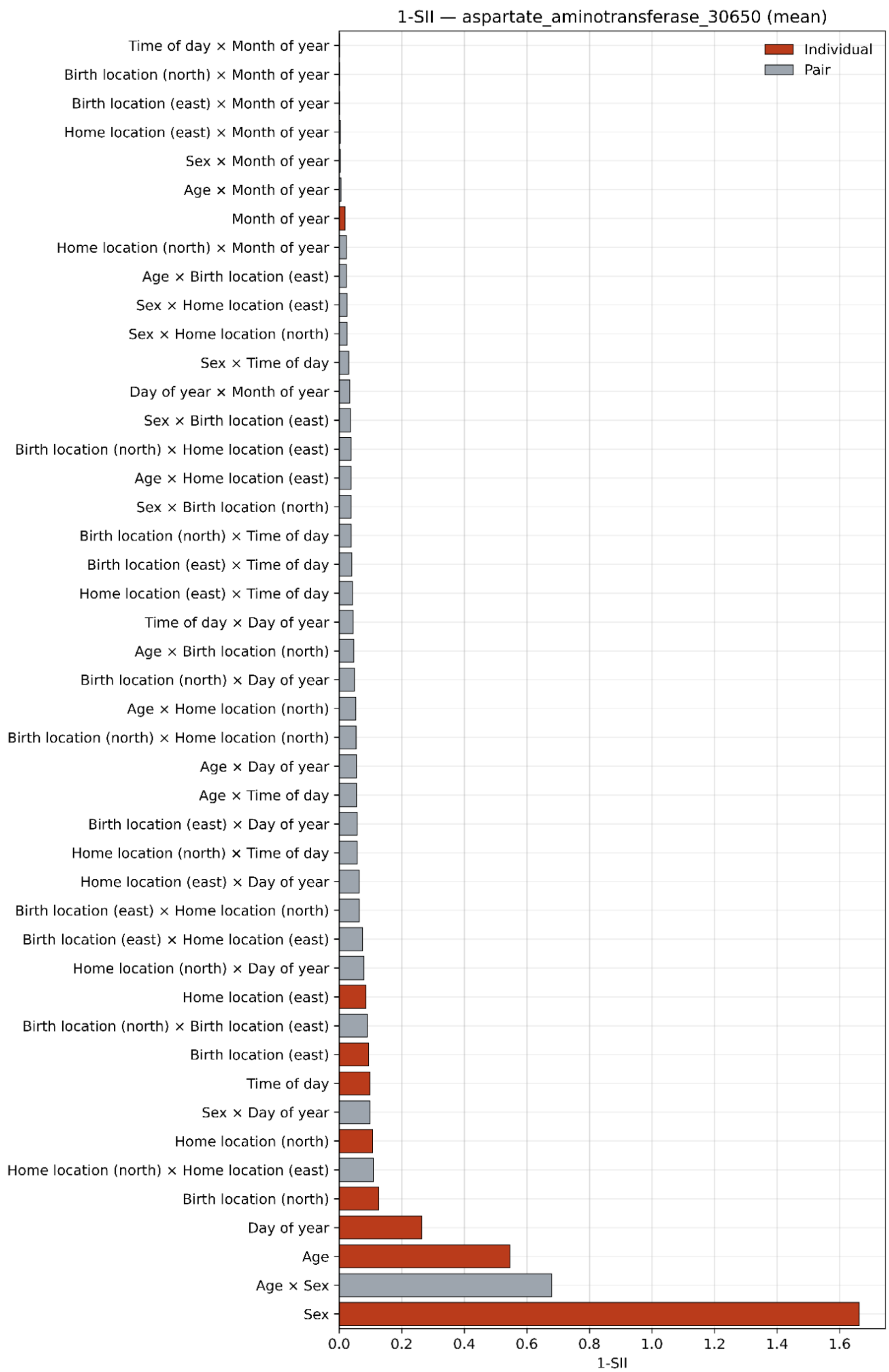

Supplementary Figure 6c

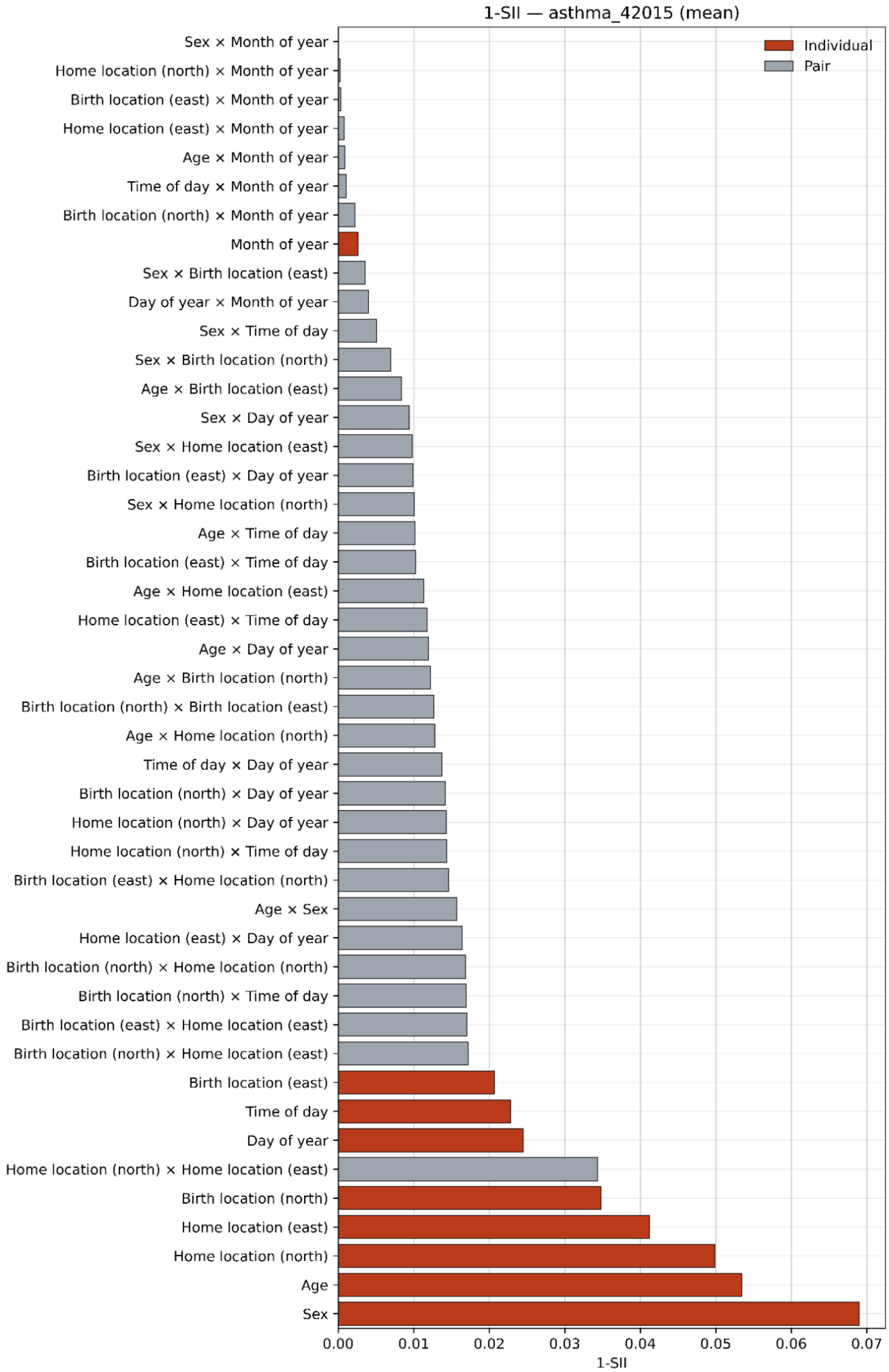

Supplementary Figure 6d

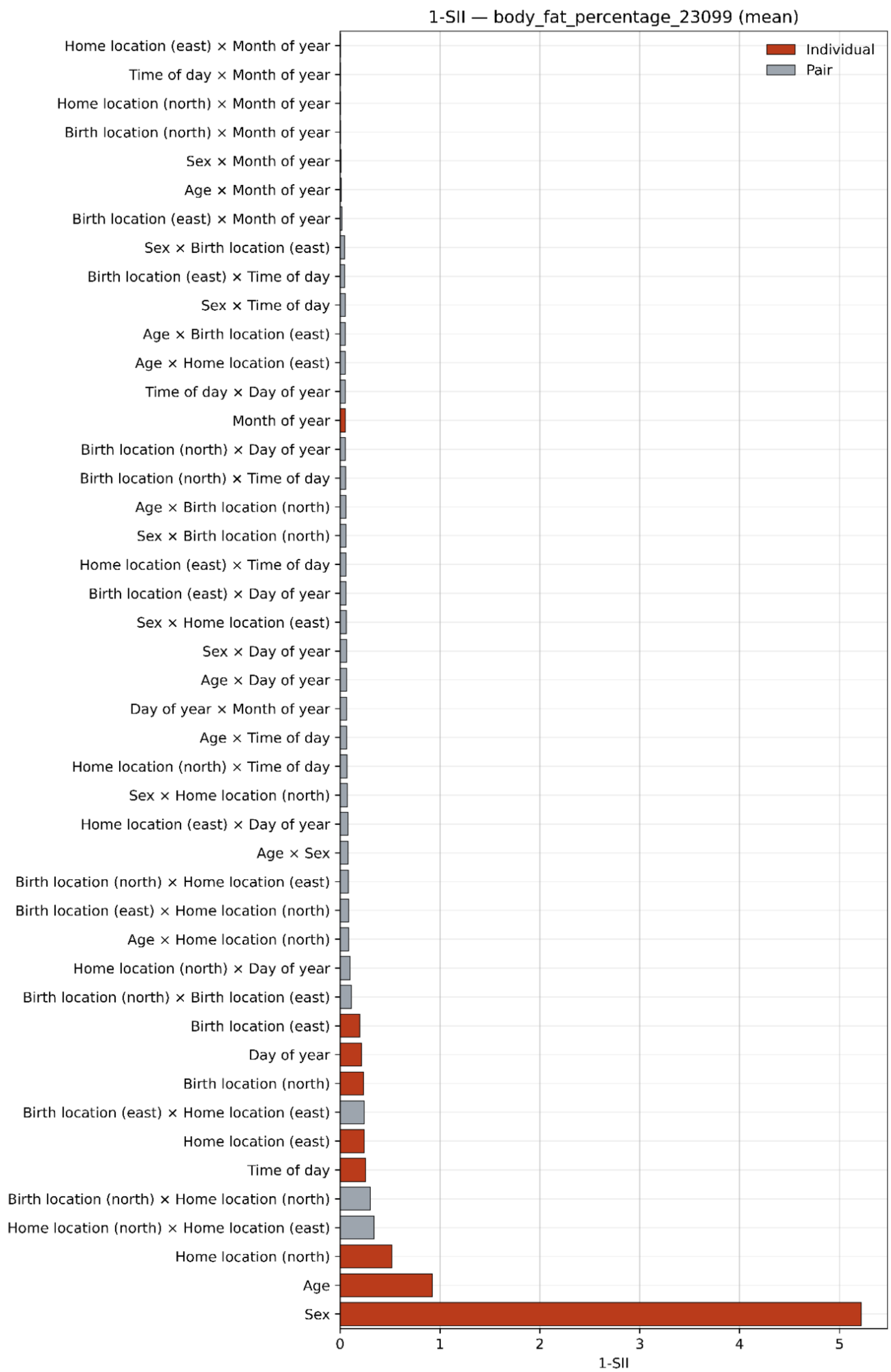

Supplementary Figure 6e

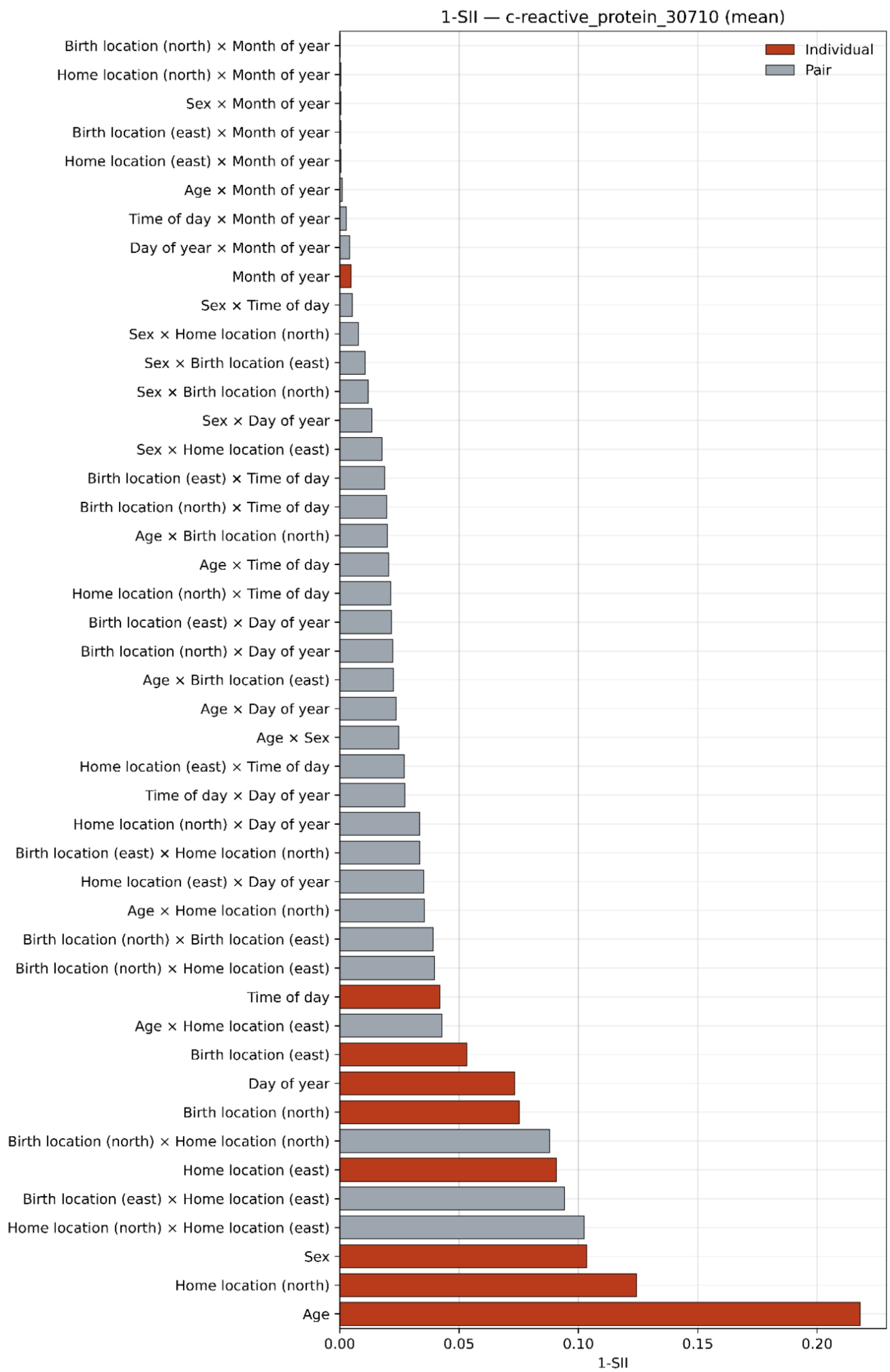

Supplementary Figure 6f

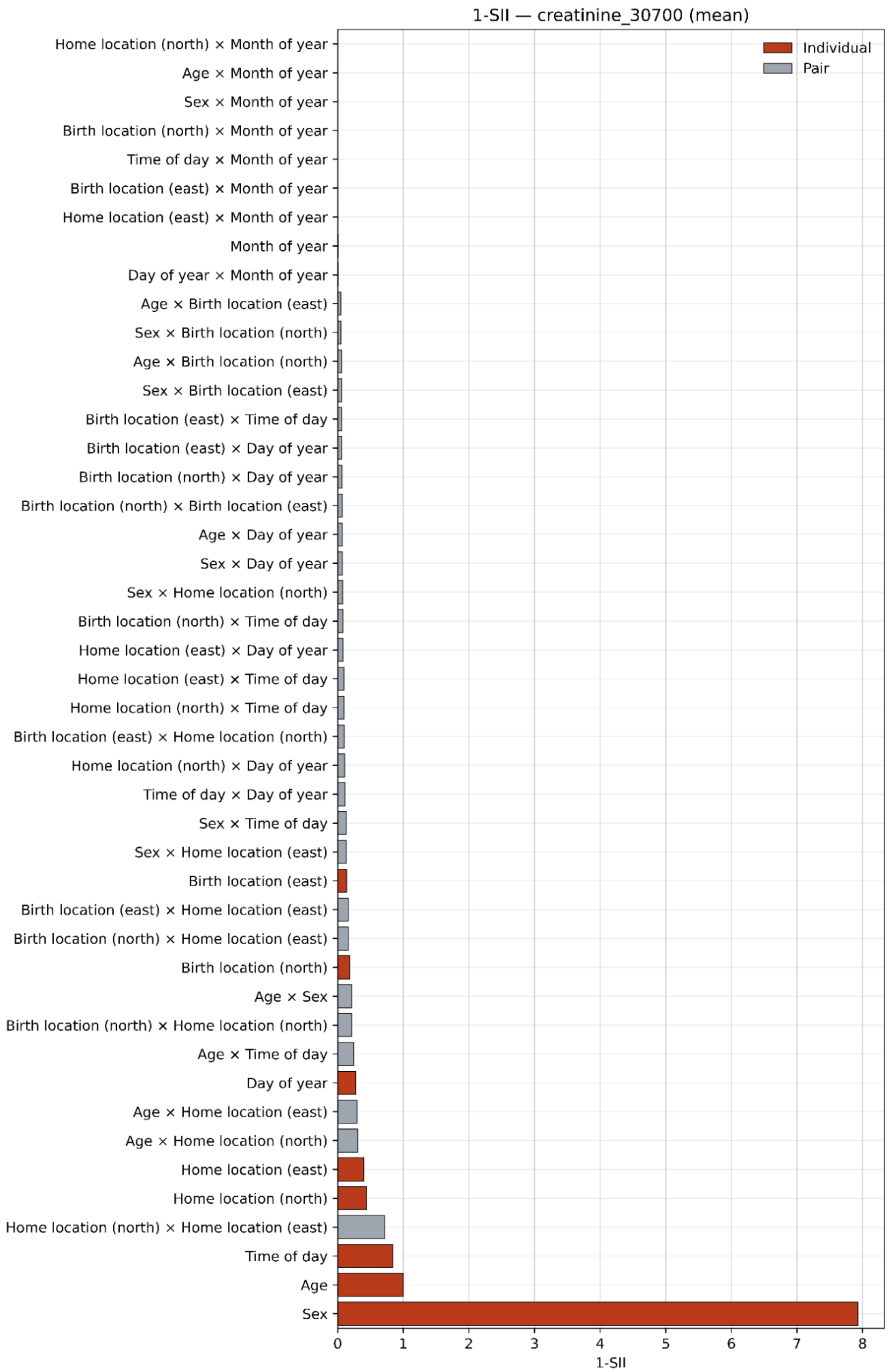

Supplementary Figure 6g

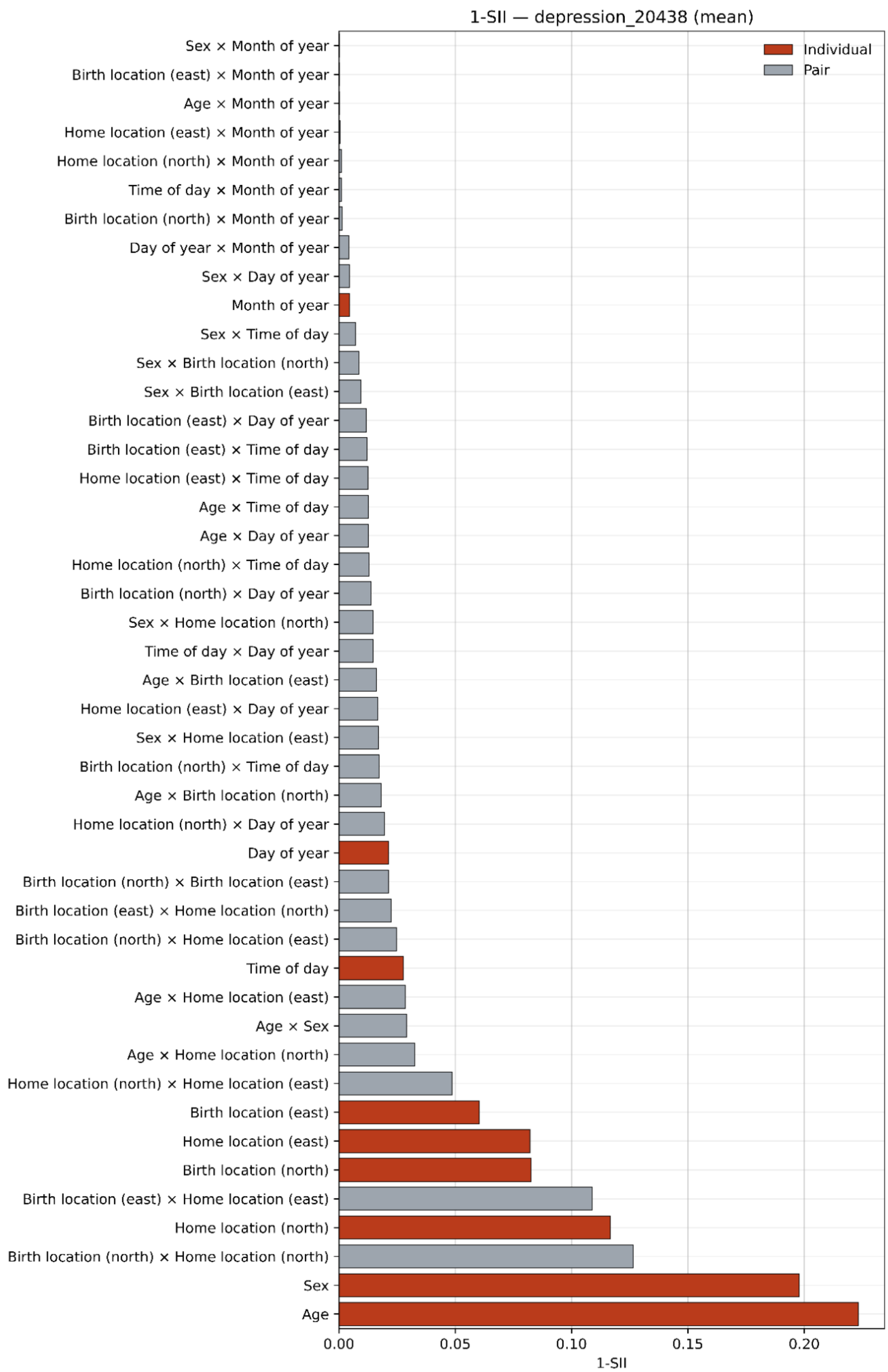

Supplementary Figure 6h

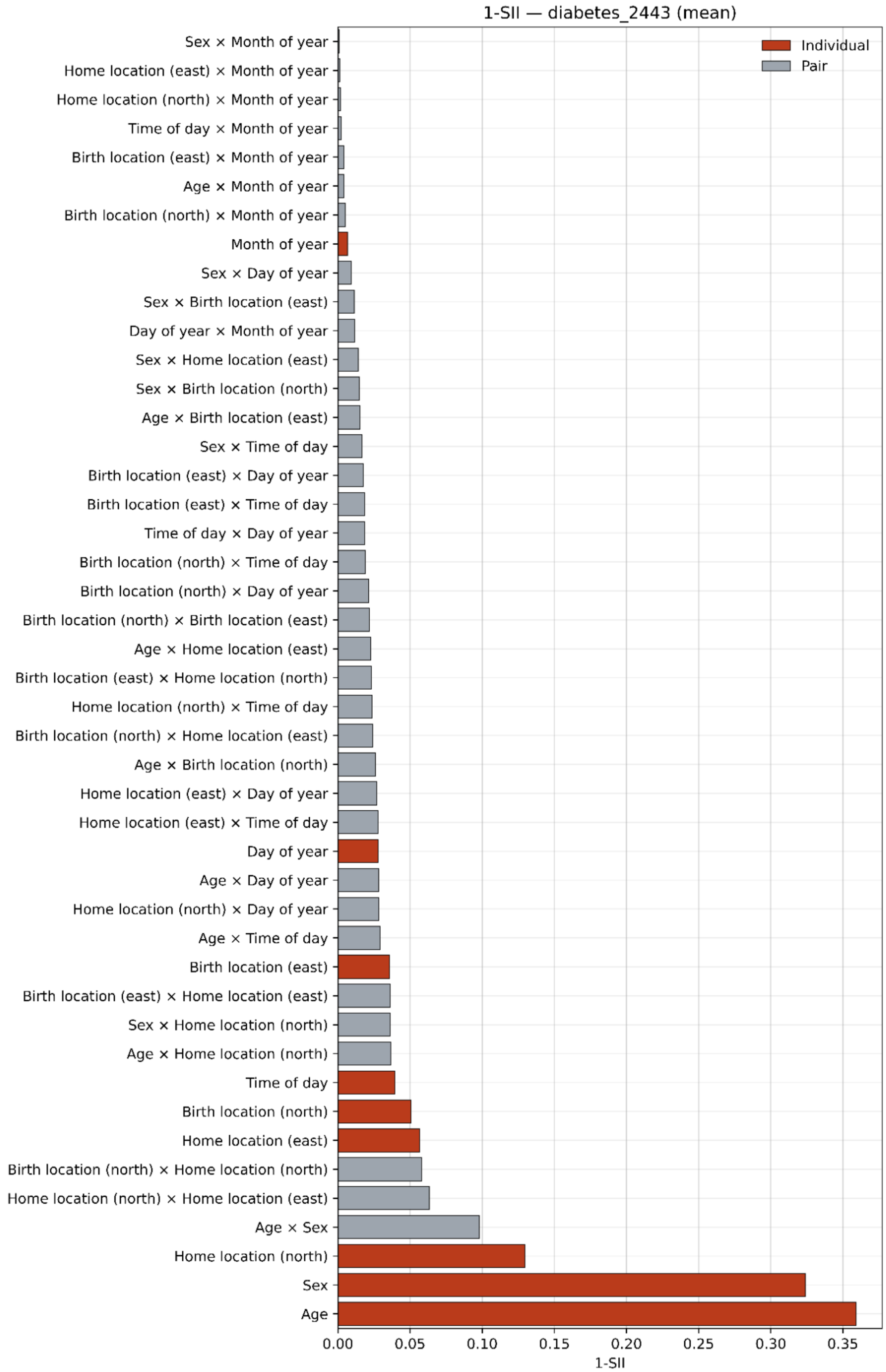

Supplementary Figure 6i

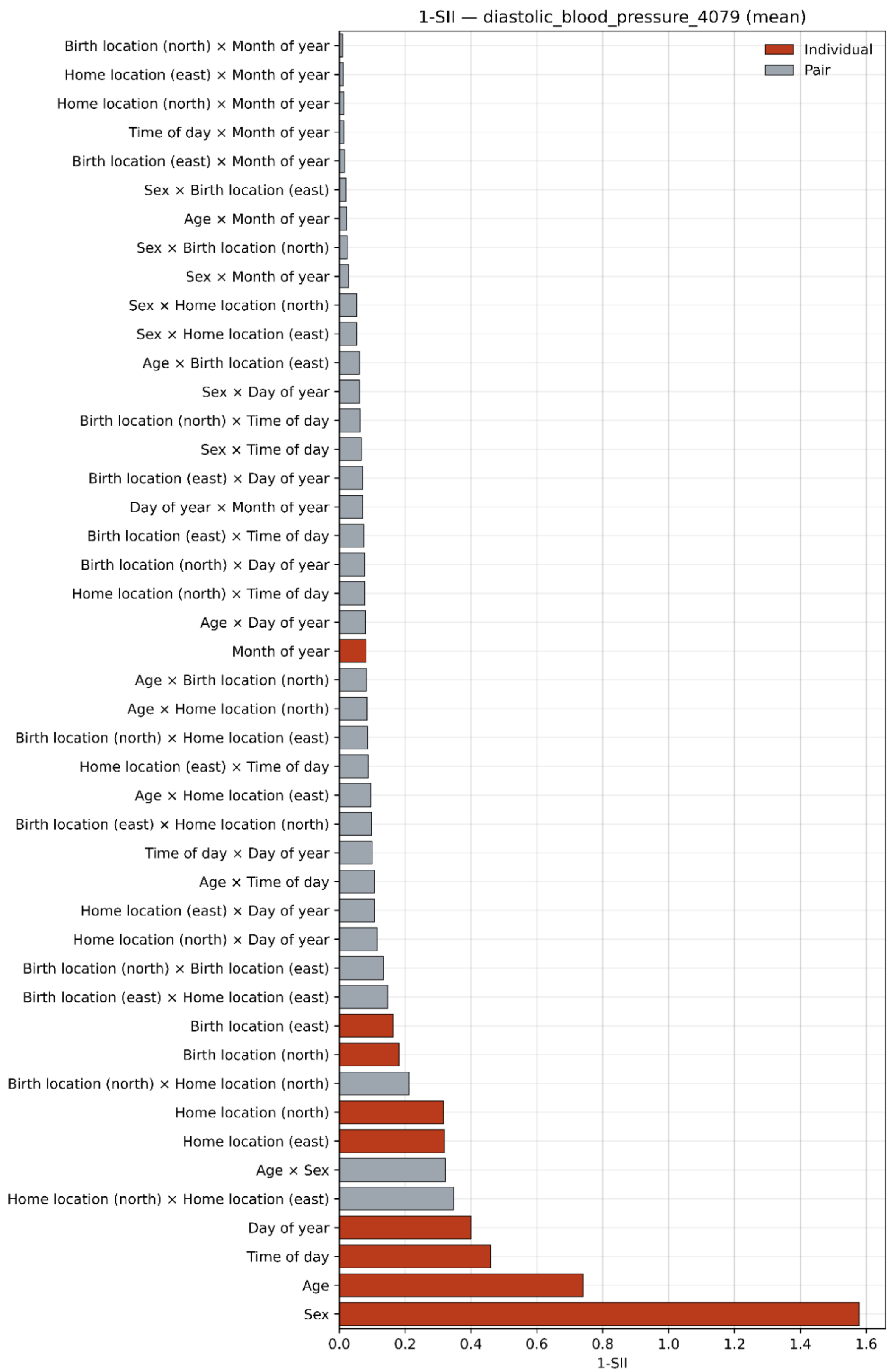

Supplementary Figure 6j

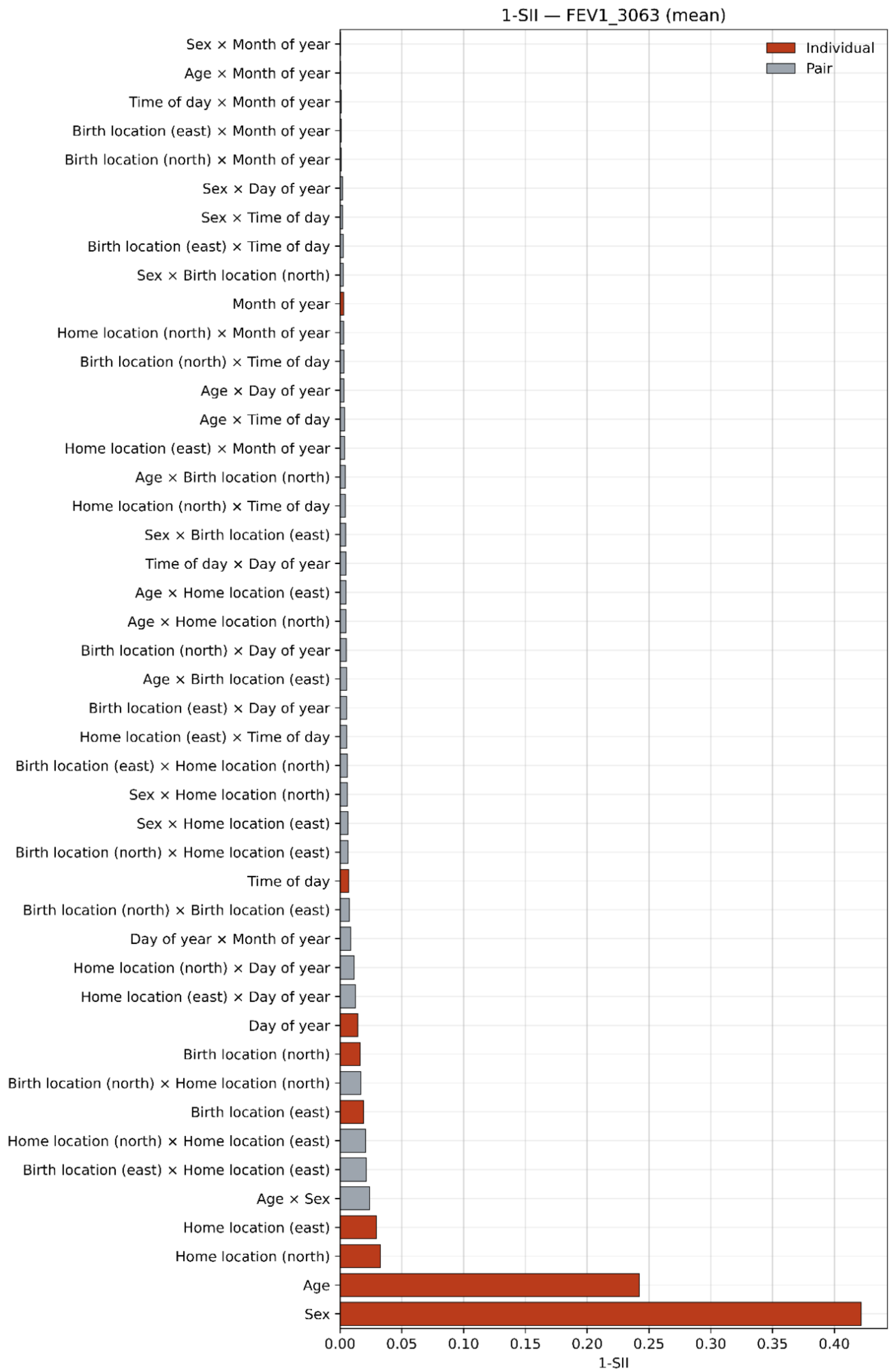

Supplementary Figure 6k

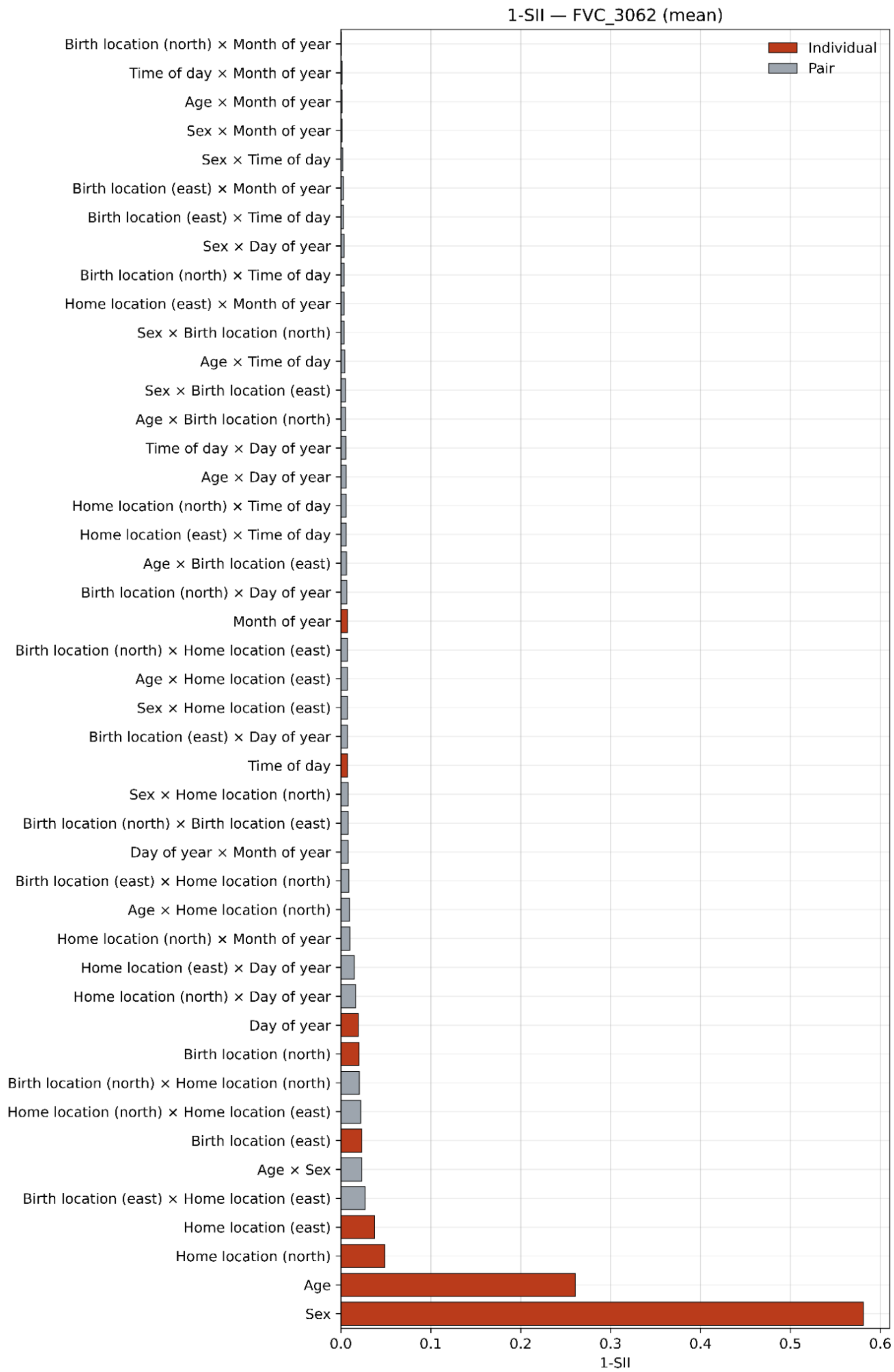

Supplementary Figure 6l

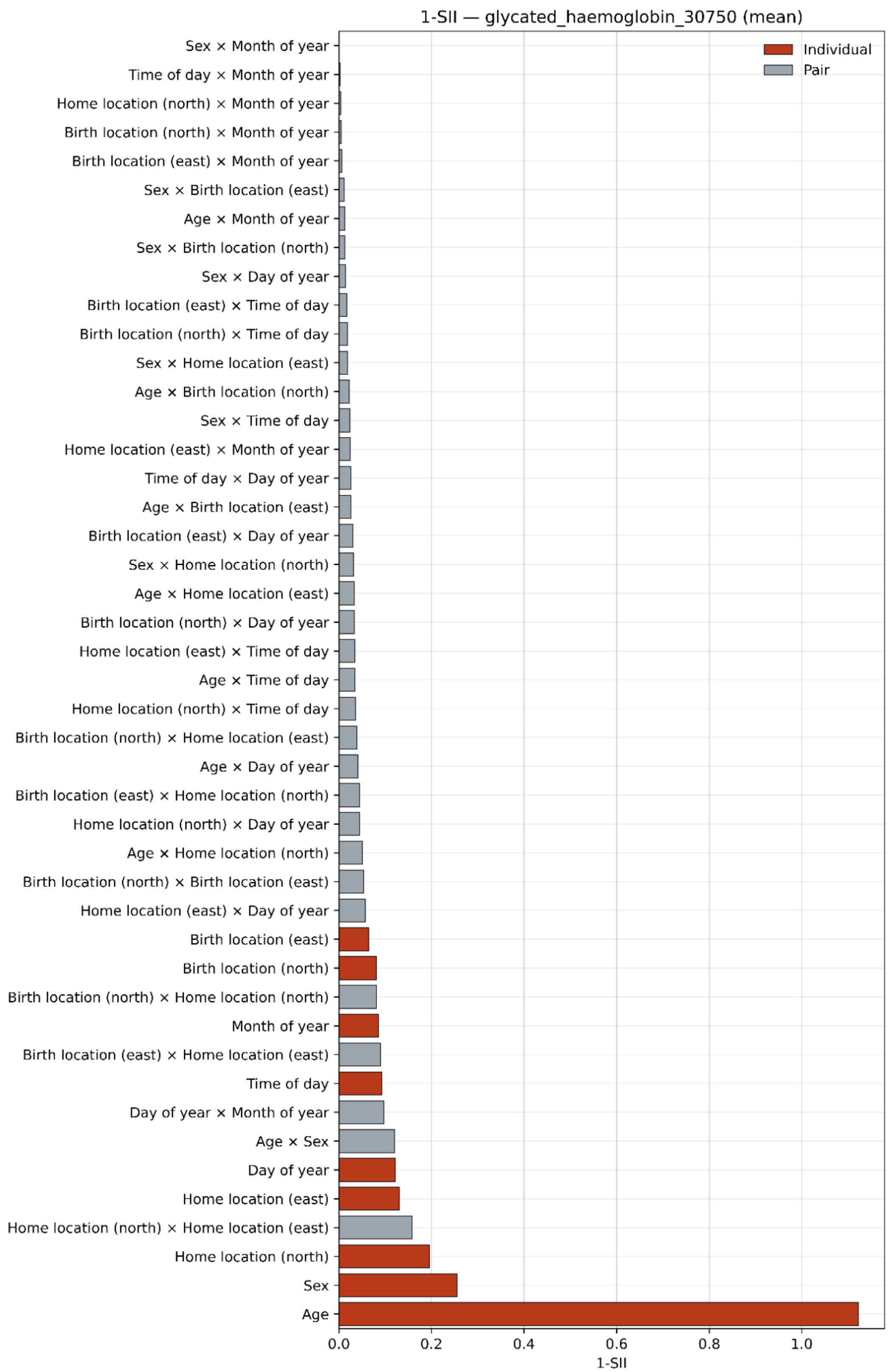

Supplementary Figure 6m

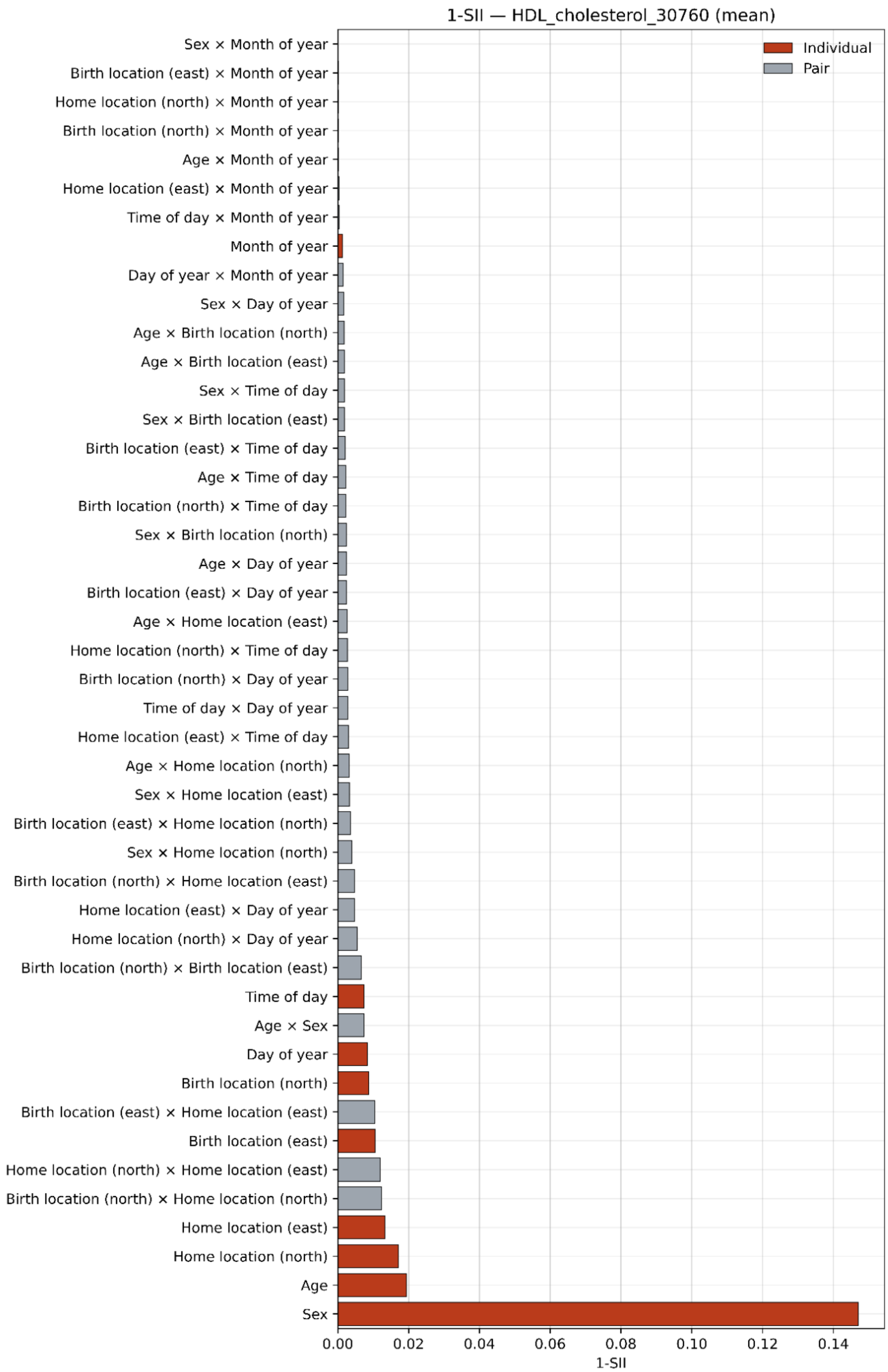

Supplementary Figure 6n

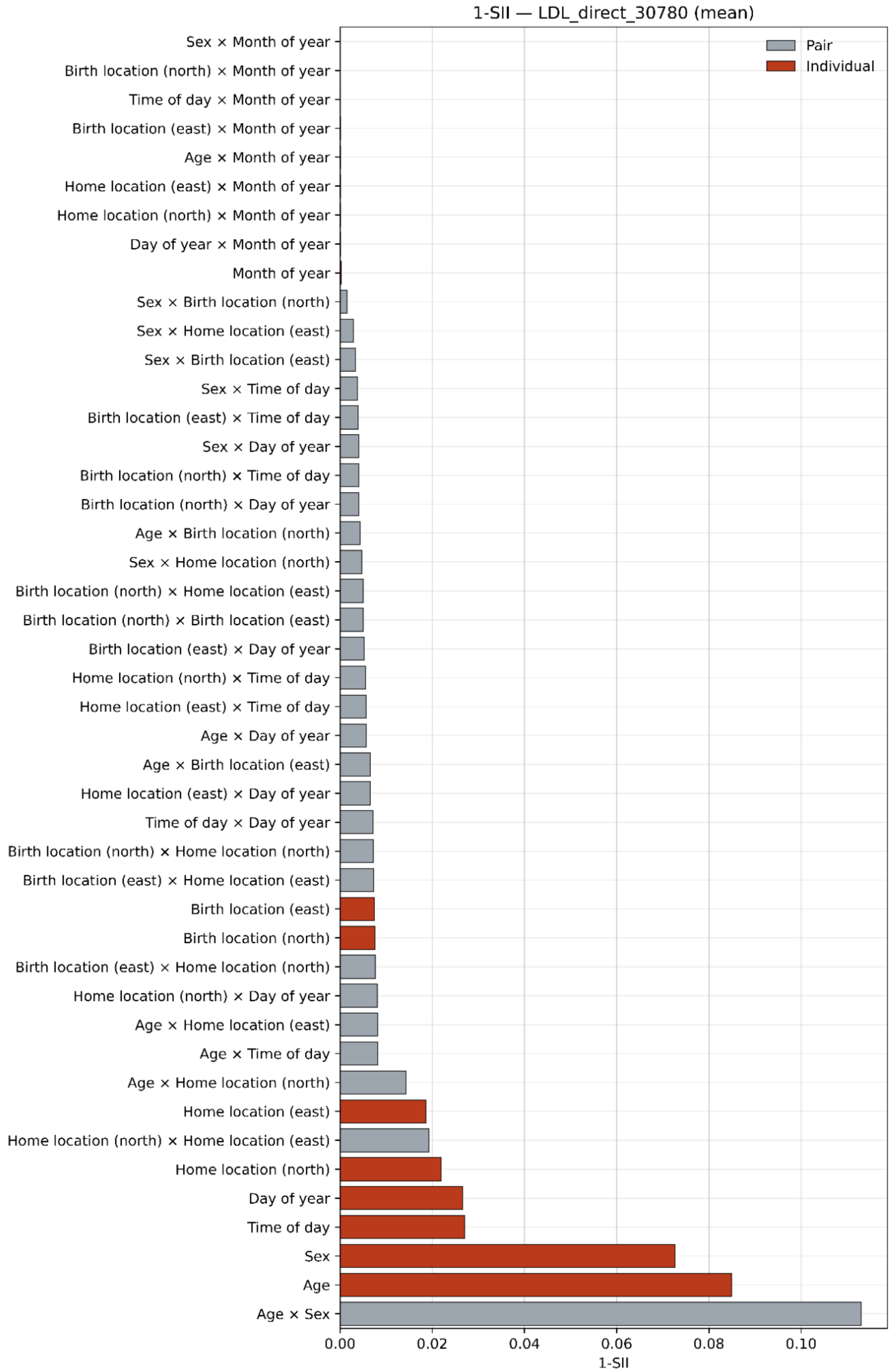

Supplementary Figure 6o

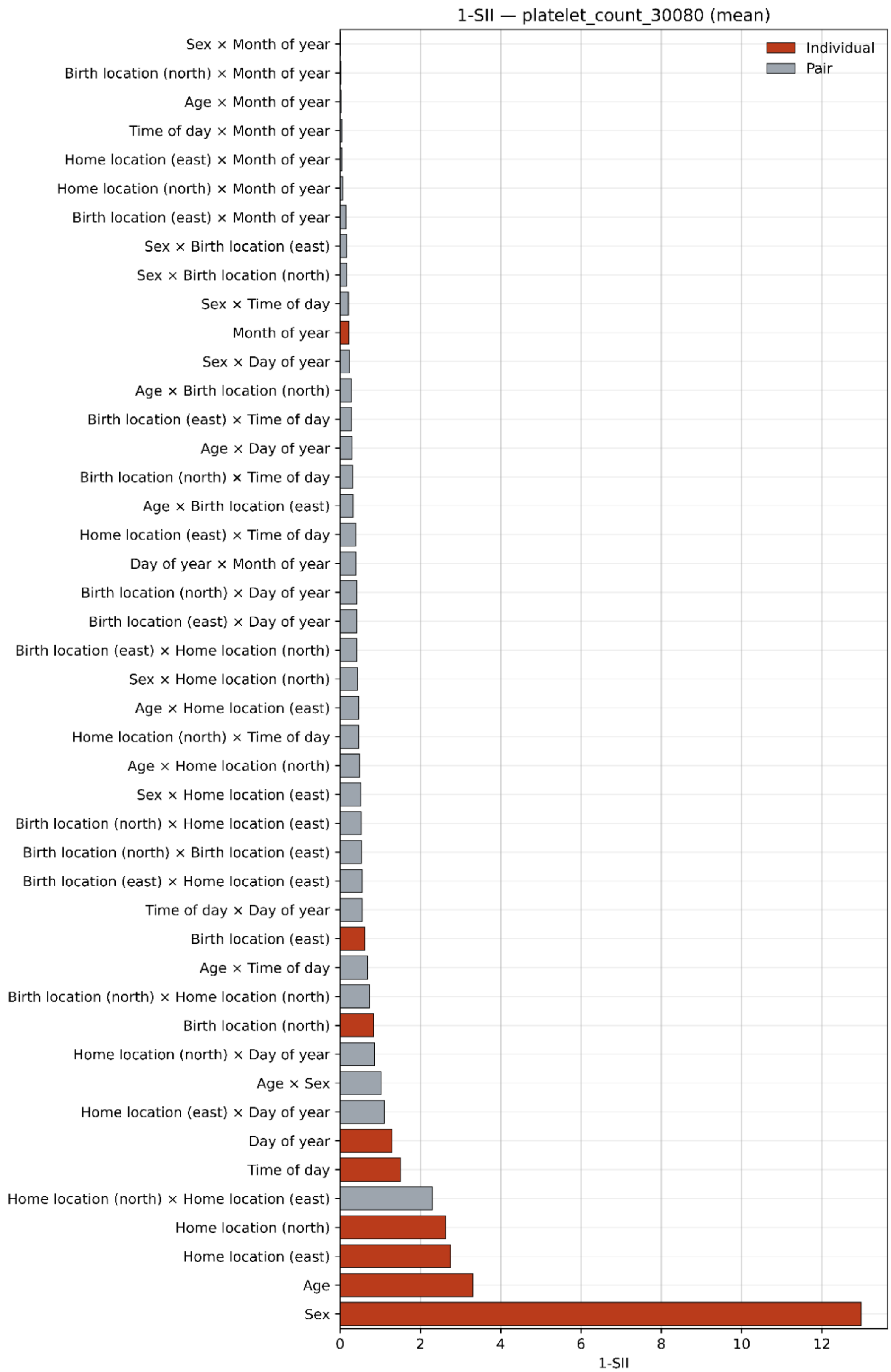

Supplementary Figure 6p

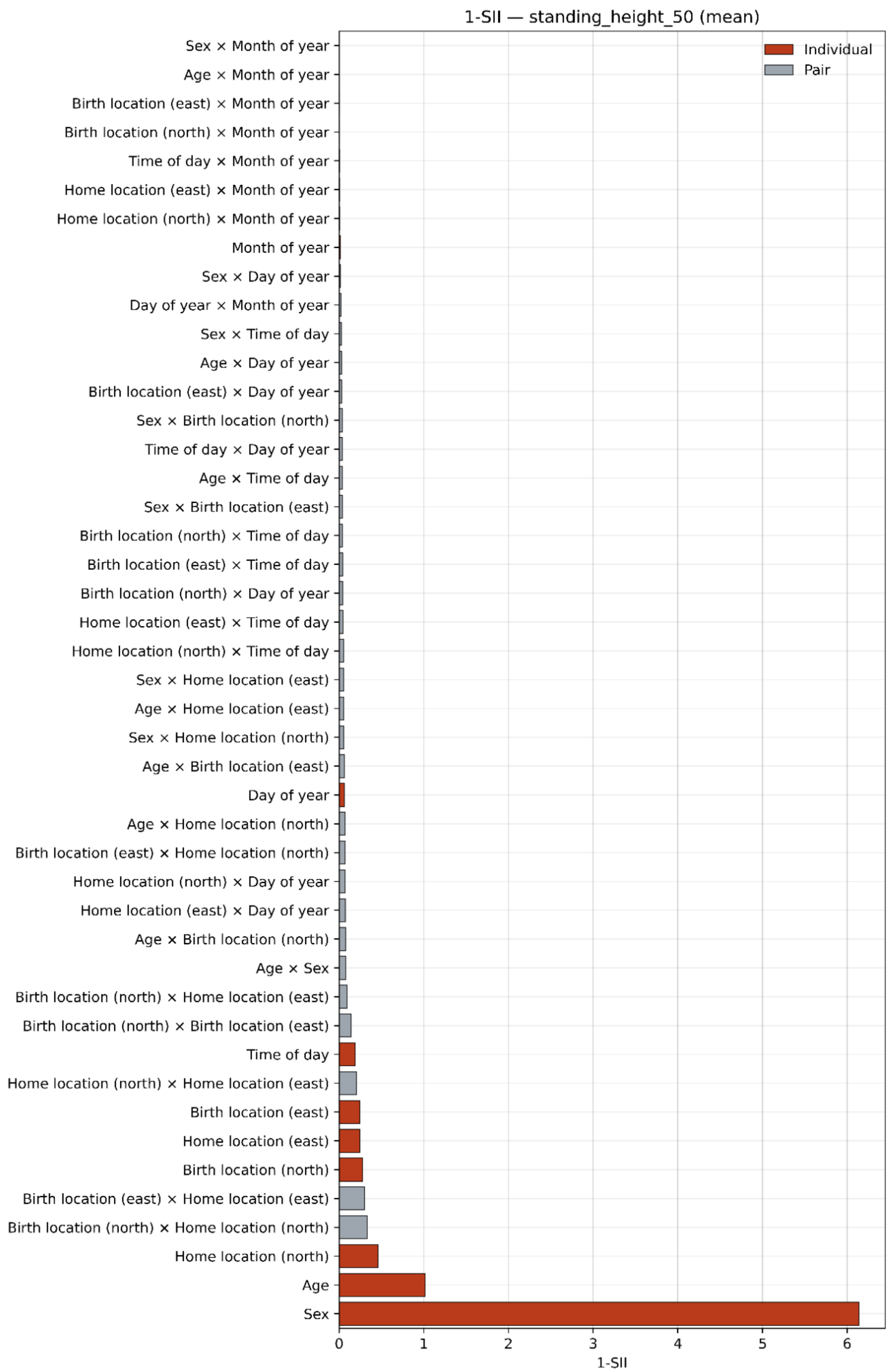

Supplementary Figure 6q

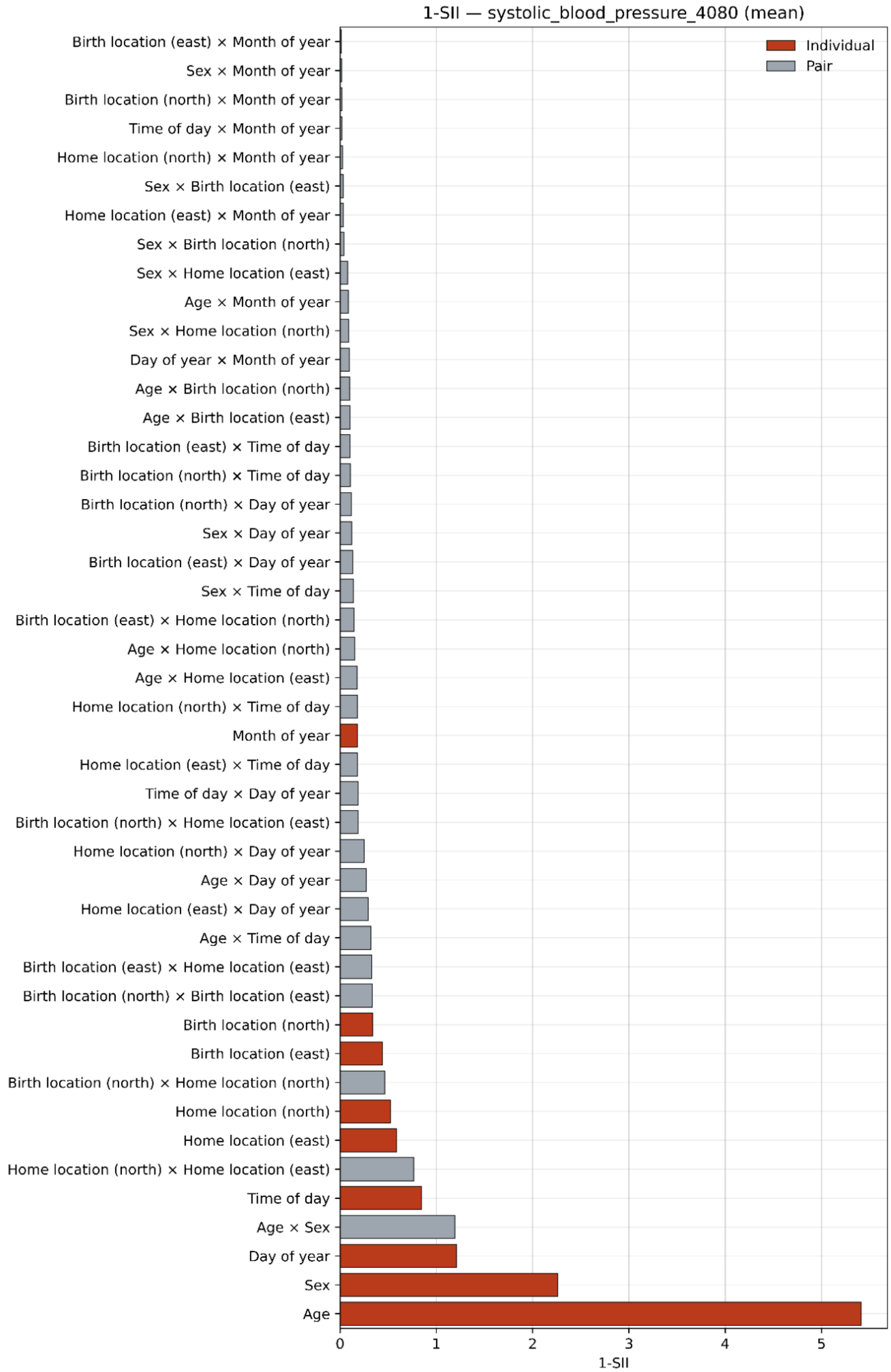

Supplementary Figure 6r

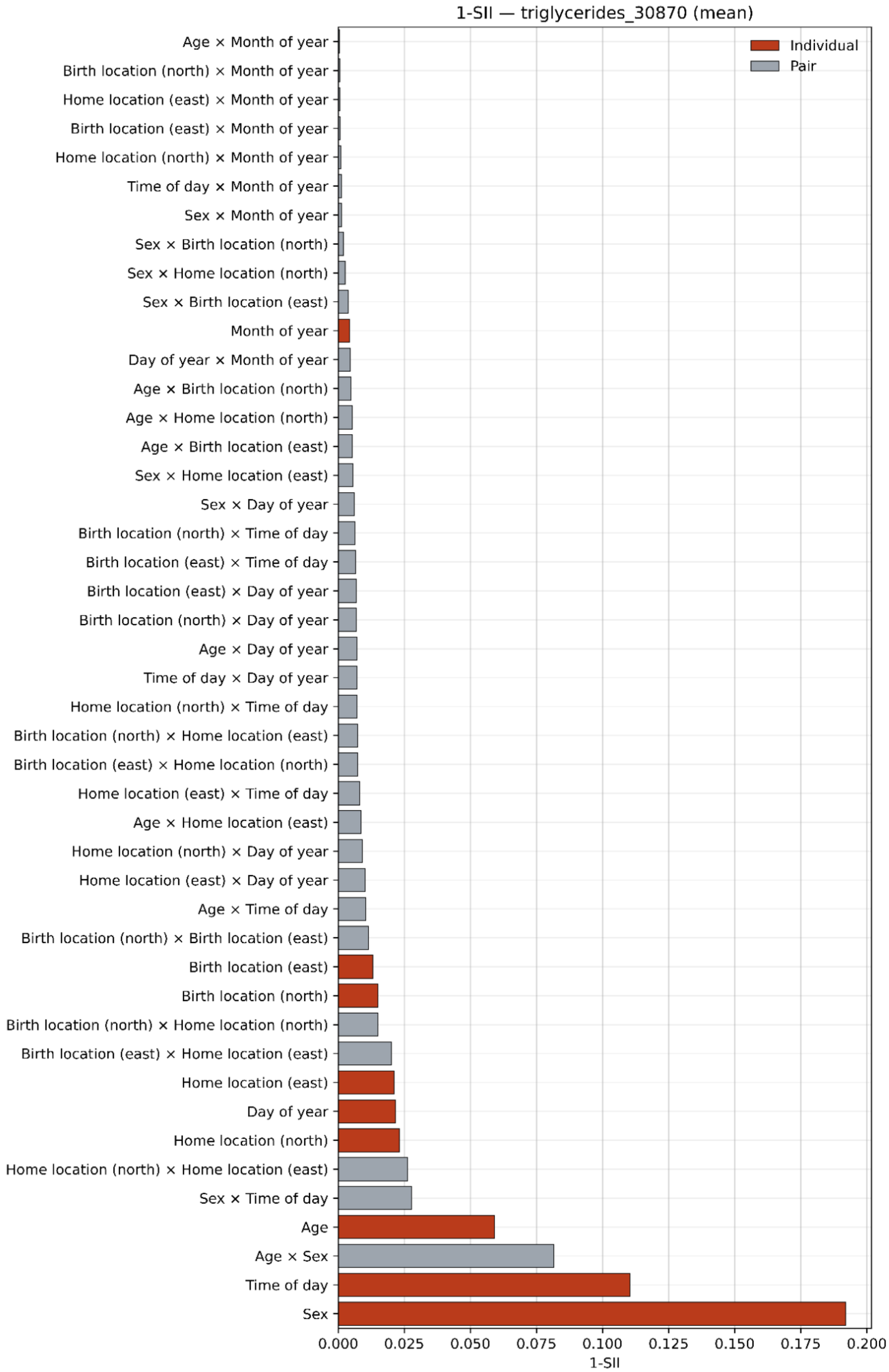

Supplementary Figure 6s

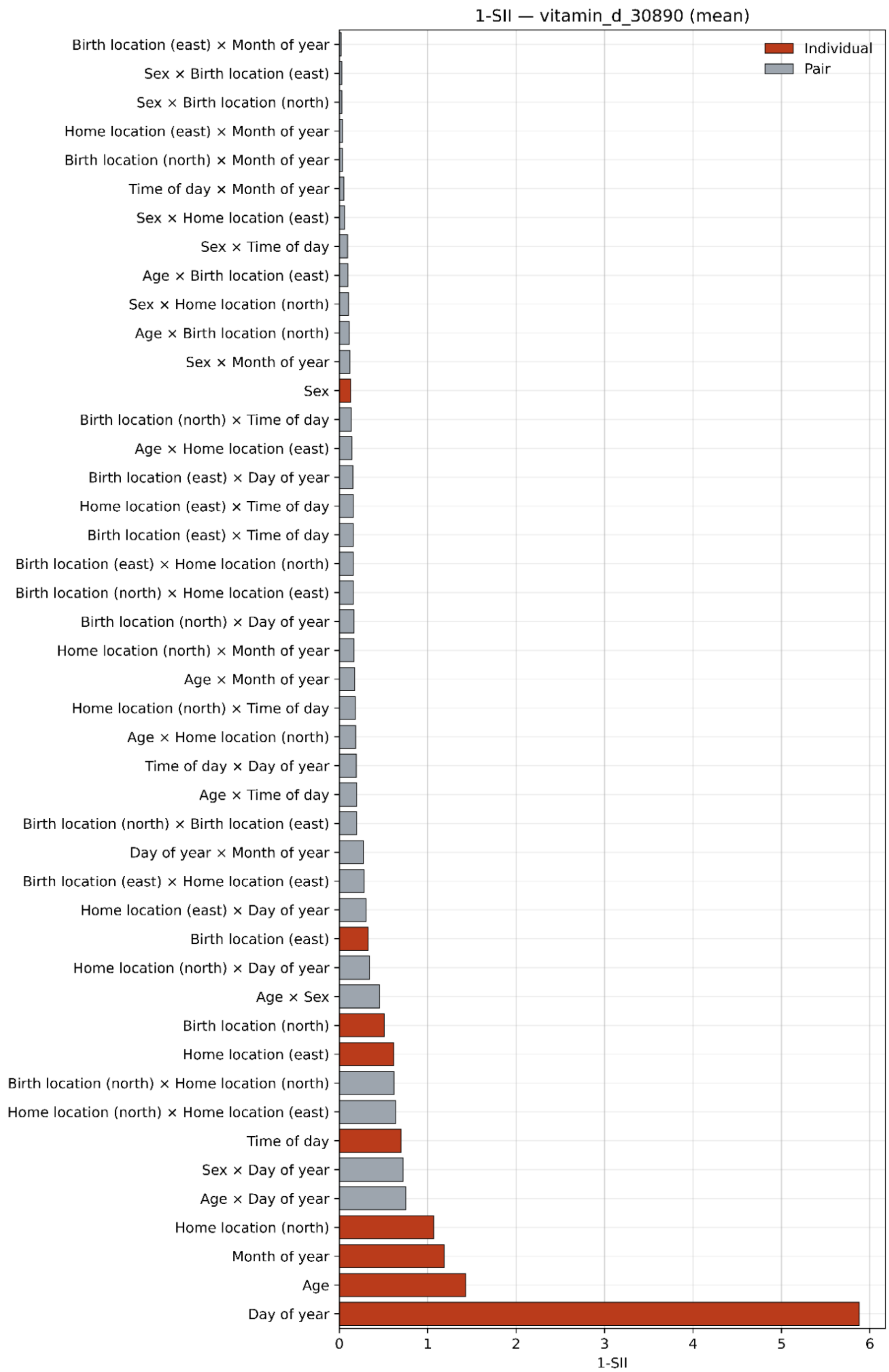

First-order Shapley Interaction Index (1-SII) values for all phenotypes. Bar plots show the mean magnitude of the 1-SII values across the test set (sample sizes provided in Supplementary Data 1) for all individual covariate features and feature pairs. Values for individual covariates are shown in red while pairs are in gray. All values can be found in Supplementary Data 10-11.

(a) Alanine aminotransferase (ALT)
(b) Aspartate aminotransferase (AST)
(c) Asthma
(d) Body fat percentage
(e) C-reactive protein (CRP)
(f) Creatinine
(g) Depression
(h) Diabetes
(i) Diastolic blood pressure
(j) Forced expiratory volume in 1 second (FEV1)
(k) Forced vital capacity (FVC)
(l) Glycated haemoglobin
(m) HDL cholesterol
(n) LDL (cholesterol) direct
(o) Platelet count
(p) Standing height
(q) Systolic blood pressure
(r) Triglycerides
(s) Vitamin-D
