# Supplementary material for: Modeling nonlinear and interaction effects of spatiotemporal and nongenetic factors improves prediction for complex traits
Source: Nat Commun. 2026 Aug 29;17:10333. doi: 10.1038/s41467-026-76154-7 (PMC13623873; doi:10.1038/s41467-026-76154-7)
Supplement: Supplementary file 4 [file 41467_2026_76154_MOESM2_ESM.pdf]

Description of Additional Supplementary Files

File Name: Supplementary Data 1
Description: Phenotype sample counts. The table contains the number of participants in the train, validation, and test splits for each of the phenotypes included in this study.

File Name: Supplementary Data 2
Description: Null model performance. Null model performance with different model variations and covariate sets. Training and evaluation was performed using 5-fold cross validation with the training set.

File Name: Supplementary Data 3
Description: Cyclic basis function null. Table for evaluating the impact of cyclic basis functions for temporal covariates on null model performance. Includes model performance in R^2 for null models with and without cyclic basis function features and two-sided Fisher-Pitman test p-value for models not being exchangeable.

File Name: Supplementary Data 4
Description: Null costs and runtime. Costs and runtimes to train null model on AWS instances via the UKB-RAP. Also, average costs by null model by model class and specific type.

File Name: Supplementary Data 5
Description: Null for non-White British. Performance of XGBoost and linear (ridge regression) null models for participants with non-White British self-reported ethnicity, as well as White British participants for comparison.

File Name: Supplementary Data 6
Description: PGS model performance. PGS model performance with different PGS methods, covariates, and with/without null models. Reported results are from evaluation on the test set.

File Name: Supplementary Data 7
Description: Null model GWAS impact. Shows changes in counts of significant GWAS hits when XGBoost null model is used with all additional covariates compared to baseline approach. P-values used to define significance are from two-sided t-tests on per-variant additive-effect coefficients from linear regressions fit with PLINK 2 --glm and are uncorrected.

File Name: Supplementary Data 8
Description: Pairwise PGS significance. Pairwise two-sided Fisher-Pitman test unadjusted p-values between baseline and alternative model.

File Name: Supplementary Data 9
Description: Neural network PGS. Comparison of PGS using XGBoost or neural network null models for phenotypes were a neural network did best in terms of MAE.

File Name: Supplementary Data 10
Description: Mean 1-SII values. Mean first-order Shapley interaction index (1-SII) values for each covariate pair across all test set participants.

File Name: Supplementary Data 11
Description: Mean SHAP values. Mean SHAP values for each covariate across all test set participants.
